# A live cell assay of GPCR coupling allows identification of optogenetic tools for controlling Go and Gi signaling

**DOI:** 10.1186/s12915-017-0475-2

**Published:** 2018-01-16

**Authors:** Edward R. Ballister, Jessica Rodgers, Franck Martial, Robert J. Lucas

**Affiliations:** University of Manchester, Manchester, UK

**Keywords:** Optogenetics, Opsin, GPCR, GalphaO, Retinal degeneration, Gene therapy, Synthetic biology, Cell signaling

## Abstract

**Background:**

Animal opsins are light-sensitive G-protein-coupled receptors (GPCRs) that enable optogenetic control over the major heterotrimeric G-protein signaling pathways in animal cells. As such, opsins have potential applications in both biomedical research and therapy. Selecting the opsin with the best balance of activity and selectivity for a given application requires knowing their ability to couple to a full range of relevant Gα subunits. We present the GsX assay, a set of tools based on chimeric Gs subunits that transduce coupling of opsins to diverse G proteins into increases in cAMP levels,  measured with a real-time reporter in living cells. We use this assay to compare coupling to Gi/o/t across a panel of natural and chimeric opsins selected for potential application in gene therapy for retinal degeneration.

**Results:**

Of the opsins tested, wild-type human rod opsin had the highest activity for chimeric Gs proxies for Gi and Gt (Gsi and Gst) and was matched in Go proxy (Gso) activity only by a human rod opsin/scallop opsin chimera. Rod opsin drove roughly equivalent responses via Gsi, Gso, and Gst, while cone opsins showed much lower activities with Gso than Gsi or Gst, and a human rod opsin/amphioxus opsin chimera demonstrated higher activity with Gso than with Gsi or Gst. We failed to detect activity for opsin chimeras bearing three intracellular fragments of mGluR6, and observed unexpectedly complex response profiles for scallop and amphioxus opsins thought to be specialized for Go.

**Conclusions:**

These results identify rod opsin as the most potent non-selective Gi/o/t-coupled opsin, long-wave sensitive cone opsin as the best for selectively activating Gi/t over Go, and a rod opsin/amphioxus opsin chimera as the best choice for selectively activating Go over Gi/t.

**Electronic supplementary material:**

The online version of this article (doi:10.1186/s12915-017-0475-2) contains supplementary material, which is available to authorized users.

## Background

G-protein signaling is central to many aspects of cellular and organismal biology. Opsins, a family of light-activated G-protein-coupled receptors (GPCRs), therefore offer the potential for optogenetic control of a wide range of processes, analogous to the optogenetic revolution enabled by ionophoric tools such as microbial channelrhodopsin, but relevant for a much broader range of cell types. Opsins are found across Eumetazoa and have members known to couple to Gα subunits in the Gi/o/t, Gq/11/14, and Gs/olf families [1, 2]. Wild-type and engineered opsins have been used as optogenetic tools for research [3–7] and as candidates for gene therapy, including for restoring vision in retinal degeneration [8–11]. Like all GPCRs, opsins are often capable of coupling to more than one G protein within their preferred family, and some couple to G proteins from different families [12, 13]. For any given application, identifying the best available opsin or engineering improved opsins requires comparative measurement of opsin activity toward the desired target G protein as well as toward undesired, off-target G proteins. As part of our ongoing investigation into the basic biology of opsins and their use as optogenetic activators for research and therapy, we sought to develop a simple, robust assay to compare the functional coupling of many opsins toward one or several diverse G proteins.

Comparing the activity of opsins, or indeed any GPCR, toward different G proteins, even within the same family, is complicated by the unique intrinsic biochemical properties and downstream signaling behavior of each G protein. For example, Gi, Gt, and Go are closely related, but their distinct guanisine triphosphate (GTP) binding properties necessitate using different conditions for experiments with purified opsin in vitro, making it impossible to compare their opsin coupling activity directly [14]. In living cells, Gi, Gt, and Go target different downstream effector proteins: Gi inhibits adenylyl cyclase, Gt stimulates some classes of phosphodiesterase, and Go acts on diverse targets in different cell types, including ion channels, protein kinase C, and small GTPases, amongst others [15, 16]. These diverse signaling events may be measured using live-cell reporters or biochemical assays. However, because they engage different downstream pathways it is impossible to attribute differences in sensitivity or response amplitude unambiguously for a given opsin to selectivity in the G-protein activation event itself. These challenges are compounded for comparisons between G proteins of different families.

One approach that has been used in both biochemical and live-cell assays to overcome this difficulty is to construct G-protein C-terminal chimeras that serve as proxies for a set of G proteins of interest [14, 17–24]. The C-terminus is the major site of Gα/GPCR interaction [25, 26], and C-terminal Gα chimeras adopt the GPCR specificity of their C-terminus, while retaining the downstream signaling activity of the main body of the Gα. Several studies have shown that many GPCRs exhibit similar patterns of activity with C-terminal G-protein chimeras relative to the different Gα subunits represented by those chimeras [14, 17–24]. Rewiring a GPCR’s coupling to different G proteins through a single downstream pathway allows direct comparison of opsin coupling to a proxy of a G protein of interest, without the need to employ fundamentally different assays for each target and off-target G protein.

**Fig. 1:**
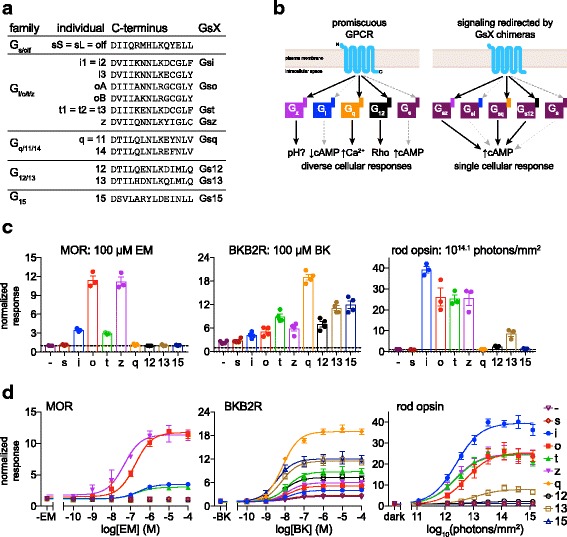
Profiling GPCR and opsin G-protein selectivity using the GsX assay. **a** C-terminal 14 amino acids of all human Gα subunits, grouped by family. Gα subunits with identical C-terminal sequences (e.g., Gq and G11) are represented with a single entry. The eight GsX chimeras used in this study are indicated at right. All GsX chimeras consist of wild-type GsS with the 13 amino acids after the final conserved aspartate replaced by the C-terminal 13 amino acids of the donor Gα subunits. In Gsi, Gso, and Gst chimeras, the -4 cysteine is replaced by serine (not shown) for insensitivity to pertussis toxin. GsX chimeras representing Gi3, GoB, and G14 were not tested. **b** Promiscuous GPCRs couple to multiple Gα subunits and activate different cellular responses. In this case, a hypothetical GPCR activates Gz, Gq, and G12 (black arrows), but not Gi or Gs (dashed gray arrows). In the GsX assay, this GPCR produces an increase in cAMP when co-transfected with Gsz, Gsq, or Gs12, but not Gsi or wild-type Gs. **c**,**d** HEK293T cells treated with pertussis toxin and transfected with Glo22F cAMP reporter and mu-opioid receptor (MOR), bradykinin receptor B2 (BKB2R), or rod opsin, without exogenous Gα (-), + wild-type Gs (s), or + GsX were stimulated with endomorphin-1 (EM), bradykinin (BK), or 470 nm light. GloSensor cAMP luminescence for each trial was normalized to pre-stimulus baseline, and the maximum cAMP fold-change post-stimulus (normalized response) within 20 mins for BKB2R and MOR, or 10 mins for rod opsin, was recorded. Replicate responses to saturating stimuli are shown in (**c**), with mean and SEM (*n* = 4, BK; *n* = 3, MOR, RHO). Stimulus–response curves (**d**) show average cAMP response +/- SEM. Fits show sigmoidal dose–response curves of the form *y*  =  *a* + *b*/1 + 10^(*c* - *x*)^ where *a* is bottom, *b* is top-bottom, and *c* is logEC50. LogEC50 and top-bottom (response amplitude) values for each GPCR/GsX combination are listed in Additional file 1. BKB2R and MOR data have been additionally normalized to account for systematic variation in response amplitude between replicates. For details of data processing, see “Methods.” BK bradykinin, BKB2R bradykinin receptor B2, EM endomorphin-1, Glo22F GloSensor 22 F, GPCR G-protein-coupled receptor, MOR mu-opioid receptor, RHO human rod opsin, SEM standard error of the mean

Adapting this idea, we developed an assay for profiling GPCR coupling that uses Gs chimeras as proxies for diverse Gα subunits. We constructed a set of Gs chimeras (GsX) in which the C-terminal 13 amino acids are replaced with eight different Gα C-terminal sequences, representing 15 of 18 human Gα proteins (Fig. 1a). Beyond the C-terminus, other regions of the Gα globular domain make minor contributions to Gα/GPCR specificity [25], but we chose not to alter these regions in the GsX chimeras to avoid unpredictable effects on GsX structure, stability, and ability to interact with Gβγ and adenylyl cyclase. In this assay, a hypothetical GPCR that couples to Gq, Gz, and G12 would activate Gs chimeras bearing a Gq, Gz, or G12 tail (Gsq, Gsz, or Gs12), stimulating adenylyl cyclase and causing an increase in cAMP (Fig. 1b). The same GPCR would not activate other GsX chimeras or wild-type Gs. Intracellular cAMP is indirectly monitored using a live-cell luminescent cAMP reporter, GloSensor™ 22 F (Glo22F), which has been extensively characterized by our lab [27] and others [28–30]. The GsX assay thus allows us to probe GPCR activity toward Gs and representatives of all other G-protein families, in real time in living cells in a multi-well format using a single biochemical pathway for direct comparison. An intrinsic limitation of using Gs chimeras as probes is that the GsX assay is applicable only to GPCRs that do not couple strongly to native Gs. Another limitation is that one can never be certain that the GsX variants are exactly equivalently active, precluding simple comparisons of a single GPCR’s activity across the GsX panel. Nevertheless, it is possible to compare GsX activity profiles between GPCRs, allowing conclusions to be drawn about differences in signaling selectivity across a group of candidate GPCRs or against a control GPCR.

Our initial application for the GsX assay is to characterize opsins with potential application in gene therapy for retinal degeneration, the progressive, irreversible loss of rod and cone photoreceptor cells. Although there is currently no cure for retinal degeneration, promising results have been obtained in animal models using gene therapy to photosensitize surviving retinal neurons using either microbial or animal opsins [8–11, 31–35]. The highest light sensitivity has been achieved by targeting animal opsins to ON-bipolar cells, a specific class of retinal interneurons [8–11, 36] that normally receive their primary signal input from a Go-coupled GPCR, metabotropic glutamate receptor 6 (mGluR6) [16, 37–39]. Maximizing opsin coupling to Go is therefore a focus for further improvement within this paradigm. Here we screen a panel of 11 Gi/o/t-coupled opsins, including two opsins that have been tested for gene therapy in animal models and several chimeric opsins that have been modified in an attempt to enhance Go coupling. We use the GsX assay to measure each opsin’s light-induced activation of proxies of Gi, Go, and Gt, and, by comparing these results against a standard (rod opsin), determine each opsin’s relative selectivity within the Gi/o/t class.

## Results

### Validating the GsX assay with MOR, BKB2R, and rod opsin

To confirm that profiles of GPCR-GsX chimera signaling replicate known profiles of interaction with native Gα subunits, we first tested the GsX chimera panel with two well-characterized non-light-sensitive GPCRs: bradykinin B2 receptor (BKB2R) and mu-opioid receptor (MOR). MOR is selective for Gi/o/t/z, while BKB2R is known to be broadly promiscuous, but with a preference for Gq [13, 40]. HEK cells transfected with a bioluminescent cAMP reporter (GloSensor 22 F, Promega), GPCR, and each GsX (Fig. 1) were stimulated with a range of concentrations of endomorphin-1 (EM, a MOR agonist) or bradykinin (BK) to construct dose–response curves, which were fit with a sigmoid function (response amplitude and EC50 values are listed in Additional file 1). The impact of any coupling to endogenous Gi (which would decrease cAMP) was minimized by including pertussis toxin (Additional file 2: Figure S1a,b). The luminescent signal from the cAMP reporter was normalized to the pre-stimulation baseline and the maximum signal post-stimulation was measured as the response for each trial.

Based on MOR’s known activity, we expect to detect a cAMP increase in this assay when MOR is co-expressed with Gsi, Gso, Gst, and Gsz, but not with the other GsX chimeras (Gsq, Gs12, Gs13, and Gs15), and not in the absence of GsX (when such a response would rely upon endogenous Gs) or transfected wild-type Gs. This is the result we see (Fig. 1c,d). We also find that EM treatment does not stimulate cAMP in the absence of MOR (Additional file 3: Figure S2a), indicating that HEK cells do not express endogenous MOR. Therefore, any response to EM can be attributed to transfected MOR activating transfected Gsi, Gso, Gst, or Gsz chimeras.

The bradykinin receptor, by contrast, is a promiscuous GPCR that couples most strongly to Gq but also to all other Gα’s, including, weakly, Gs. We, therefore, expect that BK treatment in cells expressing BKB2R would stimulate a small increase in cAMP through endogenous Gs, but that cells expressing GsX chimeras representing high-efficiency partners for BKB2R (such as Gsq) would exhibit larger responses. This is the pattern we observe (Fig. 1c,d). BKB2R stimulation produces a small increase (~1.3-fold) in cAMP in cells expressing only endogenous Gα (or transfected with wild-type Gs), compared to an 18-fold response when co-expressed with Gsq. All other GsX chimeras yielded an intermediate level of BKB2R activity, as expected for this broadly active receptor [13]. As above, BK treatment elicited no response in the absence of transfected BKB2R (Additional file 3: Figure S2b).

As our primary interest is opsins, we tested the GsX panel with human rod opsin (RHO in figure legends), which is known to couple to Gt, Gi, and Go, but not Gs. Cells transfected and treated as above were stimulated with 470 nm light using an LED array. Light potently activated Gsi, Gso, and Gst, as expected, and also Gsz (Fig. 1c,d). Gz is closely related to Gi, Go, and Gt, and is known to couple with many Gi-coupled GPCRs [41, 42]. Rod opsin did not show detectable coupling to Gsq in response to light, but, surprisingly, did exhibit detectable light responses with Gs12, Gs15, and, weakly, Gs13. To our knowledge, these are the first results indicating rod opsin can activate G12, G13, or G15. While it is unclear whether rod opsin activity toward G proteins other than Gt is significant for visual transduction in rods, this finding is informative for the general question of GPCR/Gα specificity (rod opsin is one of the most thoroughly characterized GPCRs), and for the use of this opsin as an optogenetic tool in cell types where G12, G13, or G15 are expressed.

As expected, rod opsin did not exert any light-responsive effect on cAMP in negative control experiments using endogenous or transfected wild-type Gs. This confirms that rod opsin lacks significant coupling to wild-type Gs (which would elevate cAMP). No light responses were detected with any G proteins in negative control experiments in the absence of opsin (Additional file 3: Figure S2c). These negative control results allow us to conclude that positive light responses observed with GsX chimeras are driven solely by interaction between opsin and those particular GsX constructs, specifically their C-terminal tails, since that is the only aspect of the experiment that has been varied. Importantly, although rod opsin-driven light responses (~40-fold increase in GloSensor signal when coupled to Gsi) were the largest recorded amongst this set of GPCRs, they were still substantially smaller than those produced by a natively Gs-coupled opsin, JellyOp [27, 43] (~200-fold, Additional file 2: Figure S1c). This indicates that the responses we observe in the GsX assay are far from saturating the downstream components of this signaling system, including adenylyl cyclases and the GloSensor reporter itself.

### Candidate opsins for retinal degeneration gene therapy

**Fig. 2:**
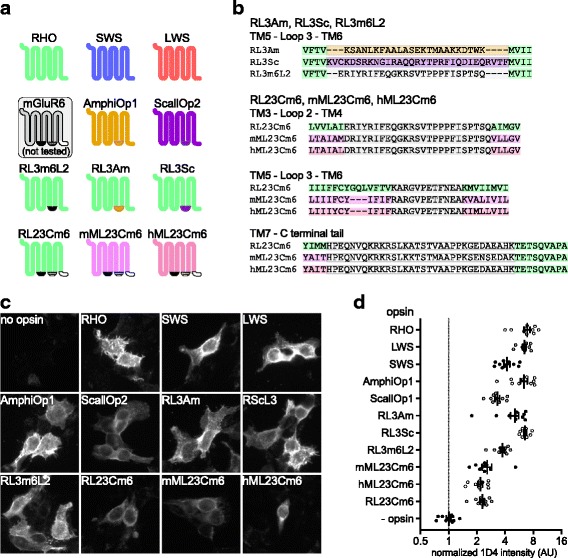
Opsins tested in this study. **a** Schematic of opsins tested (and mGluR6), illustrating intracellular regions exchanged in chimeras. RHO indicates wild-type human rod opsin, SWS indicates wild-type human short-wave-sensitive cone opsin, and LWS marks wild-type human long-wave-sensitive cone opsin. mGluR6 was not tested, but is presented to illustrate chimeric opsin construction. mML23Cm6 was previously published as Opto-mGluR6. All of the opsin constructs were tagged with the C-terminal nine amino acids of rod opsin, which is the epitope for the 1D4 monoclonal antibody. **b** Amino-acid sequences of the transmembrane helix (TM)–intracellular fragment–TM splice junctions of the opsin chimeras. **c**,**d** HEK293T cells were transfected with opsins, fixed, stained with 1D4 anti-rod opsin antibody and fluorescent secondary antibody, and imaged on a wide-field fluorescent microscope at 20× magnification. **c** Representative images of cells expressing each opsin and control. **d** Quantification of integrated fluorescence intensity (minus background) for ten randomly selected fields from each sample, normalized to mean of no-opsin control, with mean and SEM. AmphiOp1 amphioxus opsin 1, AU arbitrary units, hML23Cm6 human melanopsin/mGluR6 (mouse) triple-fragment chimera, LWS long-wave sensitive, mGluR6 metabotropic glutamate receptor 6, mML23Cm6 mouse melanopsin/mGluR6 (mouse) triple-fragment chimera, RHO human rod opsin, RL23Cm6 RHO/mGluR6 three-fragment chimera, RL3Am RHO loop 3/AmphiOp1 loop 3 chimera, RL3m6L2 RHO loop 3/mGluR6 loop 2 chimera, RL3Sc RHO loop 3/ScallOp2 loop 3 chimera, ScallOp2 scallop opsin 2, SEM standard error of the mean, SWS short-wave sensitive, TM transmembrane helix

We next applied the GsX assay to a panel of 11 putatively Gi/o/t-coupled opsins selected for potential application in gene therapy for retinal degeneration (Fig. 2a). We tested three human visual opsins: rod opsin, long-wave-sensitive (LWS) cone opsin, and short-wave-sensitive (SWS) cone opsin, and two members of the Go-opsin family, thought to be adapted for Go coupling: amphioxus opsin 1 from *Branchiostoma belcheri* and scallop opsin 2 from *Patinopecten yessoensis* [44, 45] (AmphiOp1 and ScallOp2, respectively). Our panel also includes three human rod opsin chimeras in which the third intracellular loop is replaced by either the second loop of mGluR6 (RL3m6L2), the third loop of ScallOp2 (RL3Sc), or the third loop of AmphiOp1 (RL3Am). Bovine rod opsin analogs of RL3m6L2 and RL3Sc have been previously characterized using in vitro biochemistry [14, 46]; RL3Am is original to this study. In the final three chimeras, the second and third intracellular loops and the C-terminal intracellular tail of mGluR6 have been grafted onto mouse melanopsin, human melanopsin, or human rod opsin (mML23Cm6, hML23Cm6, and RL23Cm6, respectively). The mouse melanopsin/mGluR6 chimera, termed Opto-mGluR6 in the original study, has been tested for retinal degeneration gene therapy in mice [9]. The amino-acid sequences of the chimeric fragments are shown in Fig. 2b. All opsins were constructed with the C-terminal nine amino acids of rod opsin (TETSQVAPA) as an epitope tag for the 1D4 monoclonal antibody. Immunocytochemistry confirmed that all 11 opsins were expressed in HEK293T cells (Fig. 2c,d). Rod opsin had the highest expression, while mML23Cm6, hML23Cm6, and RL23Cm6 had the lowest.

### Assays to detect light-responsive coupling to endogenous Gi, endogenous Gs, and GsX

The opsins were screened with three different assays, testing coupling to endogenous Gi [+forskolin, -PTX (pertussis toxin)], endogenous Gs (-forskolin, +PTX), and the Gsi, Gso, and Gst chimeras (-forskolin, +PTX). The range of basal (pre-flash) GloSensor cAMP levels for each combination of transfected opsin and GsX are shown in Additional file 4: Figure S3. We expected that all opsins in this set would couple to endogenous Gi and the GsX chimeras, but not endogenous Gs. In each of these assays, we measured the maximum fold-change in GloSensor luminescence relative to baseline in response to 470 nm light over a range of radiant exposures. Functionally, the opsins fell into three groups: (1) rod opsin, cone opsins, and the rod opsin loop-3 chimeras coupled to Gi, Gsi, Gst, and Gso but not Gs (as expected); (2) the opsin chimeras bearing three intracellular fragments of mGluR6 showed little activity in any assay; and (3) amphioxus and scallop opsin exhibited unexpected activity categorically different from the mammalian visual opsins. These results are discussed in detail for each group below.

### Rod opsin, cone opsins, and rod opsin loop-3 chimeras

**Fig. 3:**
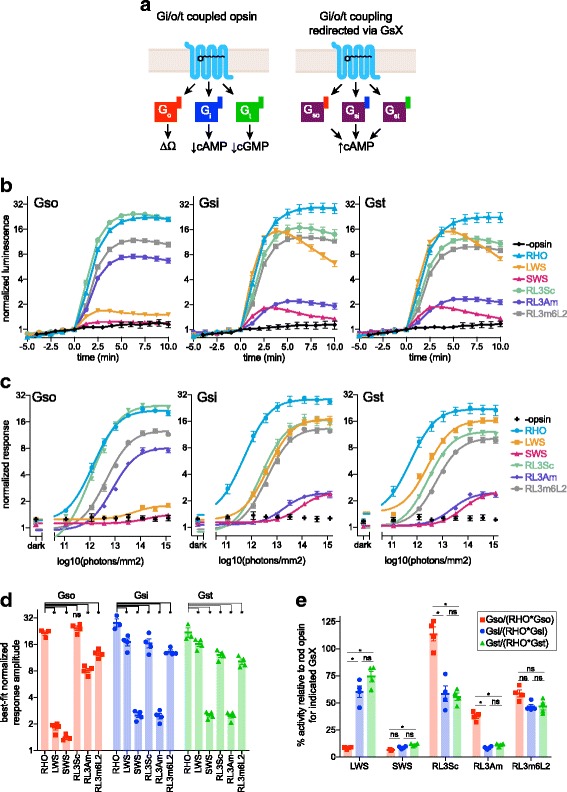
Human rod opsin, cone opsins, and rod opsin loop-3 chimeras couple to GsX. **a** Schematic of Gi/o/t-coupled opsin signaling through endogenous pathways (left) and redirected via Gsi, Gso, or Gst. Opsin is shown with all-trans-retinal, representing the active state of human visual opsins. **b**–**e** HEK293T cells treated with pertussis toxin and transfected with Glo22F cAMP reporter, opsin and Gso, Gsi, or Gst were stimulated with 470 nm light (rod opsin *n* = 3, others *n* = 4). GloSensor cAMP luminescence was normalized to baseline and light responses were calculated as detailed in “Methods.” **b** Time-course data for 10^14.1^ photons/mm^2^ flash at *t* = 0, showing mean +/- SEM at each timepoint. **c** Dose–response curves for each opsin/GsX combination, showing mean GloSensor cAMP response +/- SEM for each radiant exposure level tested. Curves are fitted to the average GloSensor response across replicates. The best-fitting parameters are listed in Additional file 7. **d** Individual trial response amplitudes from sigmoid curves for each replicate. Within each GsX, response amplitudes for the opsins were compared to that of rod opsin by ANOVA. **e** Individual trial response amplitudes in (**d**) were normalized to the average response amplitude for rod opsin for the appropriate GsX, to allow comparison of relative selectivity by ANOVA. For the ANOVA analysis in (**d**) and (**e**), α = 0.0033, based on a Bonferroni correction for multiple comparisons (initial α = 0.05, 15 comparisons total). Asterisks (*) represent comparisons that pass this threshold. Some error bars are smaller than the symbols, and in these cases the error bars are not shown. Best-fitting parameters and statistical tests pertaining to individual trials in (**d**) and (**e**) are in Additional file 10. LWS long-wave sensitive, RHO human rod opsin, RL3Am RHO loop 3/AmphiOp1 loop 3 chimera, RL3m6L2 RHO loop 3/mGluR6 loop 2 chimera, RL3Sc RHO loop 3/ScallOp2 loop 3 chimera, SEM standard error of the mean, SWS short-wave sensitive

The human visual opsins and the rod opsin chimeras in which only the third intracellular loop was substituted (RL3m6L2, RL3Sc, and RL3Am) behaved as expected for Gi/o/t-selective opsins in control assays for coupling to wild-type Gs and Gi. They exhibited no light response in the endogenous Gs assay and they suppressed cAMP in response to light in the endogenous Gi assay (Additional file 5: Figure S4, Additional file 6: Figure S5). Importantly, these opsins stimulated cAMP in response to a light pulse when co-transfected with Gsi, Gso, or Gst (Fig. 3c,d). To determine which opsin most effectively coupled to each G protein, irradiance curves were fitted for each individual trial, and response amplitudes were compared (by ANOVA) to rod opsin as a standard. When coupled to Gso, rod opsin induced an average 21.6-fold increase in the GloSensor cAMP signal over baseline, which was marginally exceeded by RL3Sc (24.6-fold), although this difference did not meet our threshold for statistical significance. The other four opsins had significantly lower response amplitudes when coupled to Gso: RL3m6L2 (12.7-fold), RL3Am (8.2-fold), LWS cone opsin (1.8-fold), and SWS cone opsin (1.3 fold). Rod opsin’s response amplitudes when coupled to Gsi and Gst (28.3-fold and 21.9-fold, respectively) were significantly greater than those of the other five opsins in this group, which ranged from 17-fold to 2.5-fold (Additional file 7). The sensitivity showed a similar pattern as the response amplitude: rod opsin had the highest sensitivity (lowest EC50 in terms of photons/mm^2^) when coupled to Gsi and Gst, and was matched in sensitivity by RL3Sc when coupled to Gso (Additional file 8: Figure S6a), and SWS cone opsin, which had the lowest response amplitudes, exhibited the poorest sensitivity.

We next sought to characterize G-protein selectivity by comparing coupling to Gso, Gsi, and Gst within each opsin. This comparison is complicated by the possibility that there may be systematic differences between Gso, Gsi, and Gst, for instance in expression, localization, or interaction with Gβγ subunits, adenylyl cyclase, or other regulatory proteins in a cell. Since it is impossible to enumerate or systematically measure all such factors that may affect G-protein performance in living cells, we controlled for potential differences by normalizing each opsin’s Gso, Gsi, ad Gst response amplitude to that of rod opsin (Fig. 3e). This allows us to compare G-protein selectivity relative to the reference opsin within this data set. There were no statistically significant differences between Gsi vs. Gst activity for any of the five opsins, indicating that none of them show a greater disparity in their Gsi/Gst coupling than rod opsin does. There were, however, striking differences in relative performance between Gso and Gsi/Gst for LWS cone opsin, RL3Sc, and RL3Am. RL3Sc and RL3Am had significantly higher responses with Gso than Gsi/Gst, whereas LWS cone opsin had much lower responses with Gso than with Gsi/Gst. Like LWS cone opsin, SWS cone opsin activity was weakest with Gso (significantly less than Gst). These results indicate that, relative to rod opsin, RL3Am and RL3Sc are selective for Gso, and LWS cone opsin is selective for Gsi and Gst (Fig. 3e). These patterns were also apparent when directly comparing response amplitudes for each opsin without prior normalization to rod opsin (Additional file 8: Figure S6b), suggesting that they are not an artifact of normalization, even considering the caveats discussed above.

### mML23Cm6, hML23Cm6, and RL23Cm6

**Fig. 4:**
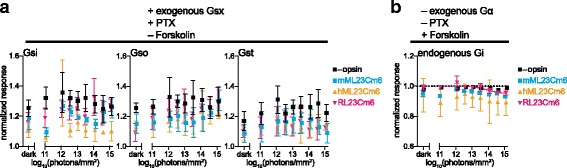
mML23Cm6, hML23Cm6, and RL23Cm6 exhibit little or no light response. **a** HEK293T cells treated with pertussis toxin and transfected with Glo22F cAMP reporter, +/- opsin, and Gso, Gsi, or Gst were stimulated with 470 nm light. Normalized light responses were calculated as above. For details, see “Methods.” None of these opsins exhibited light responses that satisfied our statistical criteria. **b** HEK293T cells were transfected with Glo22F and opsin, as indicated, and treated with 2 μM forskolin for 30 mins prior to experiment to elevate basal cAMP, in an assay for opsin coupling to endogenous Gi. Cells were stimulated with 470 nm light and the minimum GloSensor cAMP level post-flash was recorded for each trial. Only RL23Cm6 exhibited statistically significant activity, and its fitted response curve is shown. **a**, **b** Graphs show mean cAMP response +/- SEM (*n* = 3) at varying irradiance. Symbols for mML23Cm6 and RL23Cm6 have been offset slightly in the *x*-axis to aid visualization. Error bars smaller than symbols are not shown. hML23Cm6 human melanopsin/mGluR6 (mouse) triple-fragment chimera, mML23Cm6 mouse melanopsin/mGluR6 (mouse) triple-fragment chimera, PTX pertussis toxin, RL23Cm6 RHO/mGluR6 three-fragment chimera, SEM standard error of the mean

The chimeric opsins with three fragments of mGluR6 (mML23Cm6, hML23Cm6, and RL23Cm6) showed little evidence of light response when assayed for coupling to endogenous Gs (+PTX, Additional file 3: Figure S2), endogenous Gi (-PTX, + forskolin), or exogenous Gsi, Gso, or Gst (+PTX, Fig. 4). The sole exception was RL23Cm6, which produced a statistically significant light response in the endogenous Gi assay (albeit approximately tenfold less than recorded for rod opsin), but not in Gsi, Gso, or Gst assays. The lack of signaling activity for these chimeras was retained even when we employed ~100-fold more light, increased the amount of DNA transfected by 10×, or employed a different cell type (Neuro2A cells; data not shown). These findings indicate that these chimeras are much less efficient at coupling to Gi/o/t in this system than the other opsins tested here.

### ScallOp2 and AmphiOp1

**Fig. 5:**
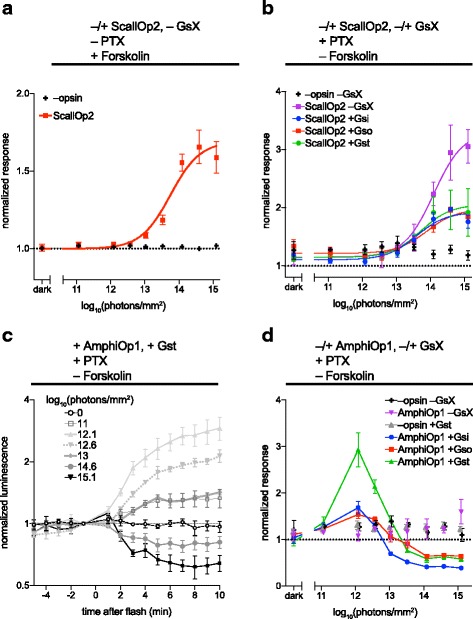
ScallOp2 and AmphiOp1 exhibit unusual signaling. **a** HEK293T cells were transfected with Glo22F, +/- ScallOp2, and treated with 2 μM forskolin for 30 mins prior to experiment to elevate basal cAMP, in an assay for opsin coupling to endogenous Gi. Cells were stimulated with 470 nm light. ScallOp2 acted to elevate cAMP in response to light in these conditions, therefore the maximum cAMP level post-flash was recorded as the response for each trial, for both + ScallOp2 and –ScallOp2 data sets. **b** HEK293T cells were transfected Glo22F, +/- ScallOp2, and GsX as indicated. They were treated with pertussis toxin (PTX) and stimulated with 470 nm light. Maximum cAMP post-flash was recorded. **c**,**d** HEK293T cells were transfected with Glo22F, +/- AmphiOp1, and GsX as indicated. They were treated with PTX and stimulated with 470 nm light as indicated. Time courses of AmphiOp1 response (+Gst) to different radiant exposures are shown in (**c**) (mean +/- SEM, *n* = 3). Because AmphiOp1 both elevated and suppressed cAMP levels after a light flash (depending on flash intensity), we recorded the largest deviation from baseline as the response for each trial. Average responses and SEM (*n* = 3) for each radiant exposure tested are shown in (**d**), with connecting lines for AmphiOp1 + GsX conditions. The symbols for –opsin/-GsX and + AmphiOp1/-GsX are offset slightly in the *x*-axis to aid visualization. Error bars smaller than symbols are not shown. AmphiOp1 amphioxus opsin 1, PTX pertussis toxin, ScallOp2 scallop opsin 2, SEM standard error of the mean

The two marine opsins, ScallOp2 and AmphiOp1, exhibited unexpected behavior. ScallOp2 stimulated cAMP in response to light in the endogenous Gs assay (+PTX, -forskolin), as well as in the endogenous Gi assay (-PTX, +forskolin) (Fig. 5a,b). ScallOp2 was the only opsin to drive an increase in cAMP signal in the endogenous Gi assay. Although the fold-change in both cases is small (+3.3-fold in the Gs assay and +1.7-fold in the Gi assay), the absolute increase in cAMP GloSensor signal in the Gi assay (~15,000 raw luminescence units or RLU) was comparable to the absolute decrease induced by rod opsin in the same assay (~18,000 RLU) or the increase induced by LWS cone opsin when coupling to Gsi (~16,000 RLU). ScallOp2’s light response was not enhanced in the presence of Gsi, Gso, or Gst, so there is no evidence that it productively couples to those constructs in this system. In summary, these data indicate that ScallOp2 drives light-dependent accumulation of cAMP via cascades endogenous to HEK cells. Ordinarily, one would assume that this represented Gs activity, but as this effect was synergistic with forskolin, some other signaling pathway may be engaged.

AmphiOp1, by contrast, showed no activity in either the endogenous Gs or Gi assays, but did affect cAMP when co-transfected with Gsi, Gso, or Gst (Fig. 5c,d). AmphiOp1 elevated cAMP when exposed to low-energy flashes, which was consistent with coupling to these chimeras as expected for Giot-coupled opsin. Surprisingly, AmphiOp1 suppressed cAMP at higher energies, suggesting that it also activates a second signaling pathway that negatively regulates cAMP. Time-course data for AmphiOp1 at stimulus intensities spanning the range tested (Fig. 5c) illustrate this biphasic response to light, which was not observed for any of the other opsins. One obvious potential origin for the additional inhibitory response is coupling to endogenous Gi in HEK cells, but the inclusion of PTX in the assay indicates that this is not the case and that the effect reflects some other signaling modality.

## Discussion

### Screening opsins for retinal prosthesis

Optogenetic activation of Go signaling in surviving ON-bipolar cells has emerged as an exciting approach for restoring vision in patients with advanced retinal degeneration. Two putatively Go-coupled opsins have been shown to restore visual responses with this approach: mammalian rod opsin and the melanopsin:mGluR6 chimera Opto-mGluR6 (termed mML23Cm6 here). An important question for this application is: Which of these established candidates has the most favorable characteristics and can efficacy be improved by targeted mutagenesis or employing an entirely different opsin (e.g., one that has evolved to couple with Go)? There has been no direct side-by-side comparison of therapeutic opsin candidates upon which to base such assessments. The GsX assay provides a useful platform for comparing opsins in living cells. While it does not address the ultimate question of in vivo utility directly, the GsX assay has allowed us to test rapidly a large number of opsins for the feature reasonably predicted to be the most important determinant of efficacy – their ability to couple to Go. An equivalent assessment of in vivo efficacy would require a significant number of animals, and therefore be much less practical in terms of time and money.

Our assay reveals that the most attractive all-around candidate for Gi/o/t-coupling is wild-type human rod opsin. While we were able readily to record Gso activity from rod opsin in our GsX assay, this was not the case for the published alternative Opto-mGluR6 (or the other mGluR6-derived chimeras tested). The lack of activity for the opsin-mGluR6 chimeras (RL23Cm6, hML23Cm6, and mML23Cm6/Opto-mGluR6) with the GsX chimeras is unexpected. Opto-mGluR6 has been shown to function in tissue culture cells when tested with patch-clamp electrophysiology, but only with sustained illumination at light levels ~100-fold greater than we used [9]. One possible explanation for the lack of activity in our assay is poor protein expression. Although we transfected an equivalent amount of plasmid DNA for all opsins, these chimeras had the lowest expression when probed by immunofluorescence. However, it is likely that chimera design also contributes. These chimeras all replace the N-terminal half of the second intracellular loop with a sequence from mGluR6. This region of rod opsin and melanopsin contains the highly conserved (D/E)RY motif, which is important for G-protein-coupling activity in many GPCRs. In the three-fragment chimeras tested here, the chimeric substitution replaces (D/E)RY with a sequence containing an intervening isoleucine: (D/E)RIY. Literature precedent indicates that rod opsin does not tolerate substitutions in this region of its second intracellular loop [46]. In light of our negative results in tissue culture cells, it is interesting that Opto-mGluR6 functions when expressed in bipolar cells of transgenic mice [9]. It is possible that ON-bipolar cells are better able to express the opsin, or that they express accessory factors that enable more efficient coupling or downstream signal amplification. Our results indicate that opsin chimeras with multiple fragments of mGluR6 are substantially less active than wild-type opsins, and highlight the need for direct comparisons between therapeutic candidates in like-for-like assays.

We tested four chimeras of rod opsin (RL3Sc, RL3Am, RL3m6L2, and RL23Cm6). In almost every assay, these chimeras had lower sensitivity and response amplitude than rod opsin (RL23Cm6 substantially so). The exception was RL3Sc, which had a higher response amplitude than rod opsin with Gso (but not Gsi or Gst). RL3Sc and RL3Am gained selectivity for Go (relative to the parental rod opsin), but for RL3Am, this came at the cost of a 2.5-fold lower response amplitude (Additional file 7). These results show that it is possible to alter opsin behavior for a desired characteristic (in this case Go selectivity), but suggest that, in general, chimeric opsins are less active than their wild-type models. Determining whether engineered opsins can exceed wild-type levels of activity toward an arbitrarily selected target G protein will require systematic testing of a large number of candidate constructs. The GsX assay provides an attractive platform for this effort, since it makes it possible to test several opsins in parallel, with different G-protein partners, to determine if chimeric modifications enhance or impair opsin function generally, and with specific G-protein targets.

### Gi- and Go-selective optogenetic tools

One salient output of the GsX assay is the finding that, compared to rod opsin, the internal control for this analysis, cone opsins appear to strongly discriminate against Go, whereas the RL3Am chimera is selective for Go. To our knowledge, this is the first evidence that cone opsins are selective against Go. These results provide a useful reference for exploring opsin-G-protein selectivity within the Giot family. Moreover, LWS cone opsin and RL3Am could be valuable optogenetic tools for selectively controlling Gi and Go, respectively. Gi is ubiquitously expressed in human cells and regulates a wide variety of processes through a common mechanism of suppressing cAMP [47]. Go is especially interesting because it is the most abundant Gα in the central nervous system, being 5–10 times more abundant than Gi and comprising up to 1% of membrane proteins in the brain [15]. Despite this, its functions in the brain are not well defined. Optogenetic tools to activate Gi and Go selectively would be useful for unraveling their relative contributions to physiological processes in the brain and elsewhere.

### ScallOp2 and AmphiOp1

Although ScallOp2 is the founding member of the Go class of opsins, which are named for their presumed Go-coupling activity, this assignation is based upon co-expression with this G protein in its native environment [45], rather than direct evidence of Go-coupling ability from biochemical or cellular assays. Contrary to our expectations, ScallOp2 exhibited light-activated stimulation of cAMP when tested in conditions designed to detect coupling to Gs (+PTX, -forskolin) or native Gi (-PTX, +forskolin). To our knowledge, this is the first report of any functional activity for ScallOp2, either in vitro or in cells. Signaling in the presence of PTX indicates that ScallOp2 is capable of coupling to at least one pertussis-toxin-insensitive pathway, that is, a G protein other than Gi, Go, or Gt, or some G-protein-independent effector. A light-dependent increase in cAMP is consistent with coupling to Gs. However, in absolute terms, this response is very small in the absence of forskolin but large in the presence of forskolin, which suggests that ScallOp2 acts on cAMP via a mechanism synergistic with forskolin-induced adenylyl cyclase activity. One possibility is light-dependent inhibition of a cyclic nucleotide phosphodiesterase. Another is that ScallOp2 stimulates endogenous Gz in HEK cells [48] in its dark state and is turned off by blue light. Gz is a pertussis-toxin-insensitive G protein that is capable of inhibiting adenylyl cyclase [42], therefore light-dependent loss of Gz activity could enhance cAMP in a manner synergistic with the effects of forskolin.

AmphiOp1 has been shown to interact with Gi in vitro in a light-dependent manner [49]. If that were its only activity, we would expect it to suppress cAMP in the Gi assay and to stimulate cAMP when co-transfected with Gsi, Gso, or Gst. The first surprising aspect of AmphiOp1 behavior is that it does not have any detectable effect on cAMP in the endogenous Gi assay, indicating that the observed light-dependent binding to Gi in vitro need not translate to productive catalytic activation in living cells. On the other hand, AmphiOp1 does stimulate cAMP in response to light at low irradiance when co-expressed with Gsi, Gso, or Gst indicating that this opsin can have activity via these pathways. However, in a second surprising result, at higher irradiance, AmphiOp1 suppresses cAMP in the same experiment. This biphasic behavior suggests that at high irradiance, AmphiOp1 activates a cryptic second pathway that suppresses cAMP below baseline, to an extent comparable to that of rod opsin coupled to endogenous Gi. This second pathway must be pertussis toxin insensitive, and could involve Gz, or other effectors altogether.

Although these two marine opsins are not strong candidates for gene therapy for retinal degeneration, these results confirm that they are both capable of signaling in human cells and that they do so via unusual mechanisms. There is an emerging body of evidence that G-protein signaling pathways are capable of crosstalk [50–53], which may be involved in the unusual signaling of these marine opsins. We hope that these results will motivate further exploration of their signaling activity, both to elucidate their function in their native contexts and to explore potential utility as optogenetic tools.

### The GsX assay

The work presented here validates the GsX assay for high-throughput screening of opsin coupling activity toward diverse G-protein partners. Gs- or GsX-driven cAMP signaling evolves on a timescale of minutes, which is much slower than ion channel or Ca^2+^ responses, but much faster than transcriptional responses. This allows a complete experimental cycle of baseline, stimulus, and response to be executed within 15 mins for a full 96-well plate using a standard luminescent plate reader, without the addition of extra reagents such as forskolin. Using the GsX assay, it is feasible to test >1000 wells in a workday, thus bringing screens involving hundreds of opsins within reach.

The GsX assay uses living cells, which has practical advantages over in vitro biochemistry. It is not necessary to laboriously purify each opsin, and opsins are tested in an intact cell membrane. In its use of C-terminal Gα chimeras, the GsX assay is conceptually similar to a system in which GPCRs are tested using G15 C-terminal chimeras to trigger a Phospholipase-C -driven pathway that terminates with proteolytic cleavage of transforming growth factor (TGF)-β and shedding of its extracellular domain, which is detected by immunoassay [22]. One important distinction is that the GsX assay utilizes a live-cell reporter, which allows continuous real-time monitoring, thus facilitating kinetic analysis, whereas the TGF-β shedding assay relies on immunoassays as an end-point readout, so kinetic information must be inferred by collecting end points across a time series, which decreases throughput. As with all assays that monitor second messenger levels, response kinetics in the GsX assay reflect the balance of competing enzymatic activities, in this case adenylyl cyclase and phosphodiesterases, and must be interpreted with this in mind [47].

One of the complications of a live-cell assay, relative to in vitro biochemistry, is that it is not feasible to measure the concentration of every relevant protein or their precise affinities and catalytic activities. Since the GsX chimeras were constructed with the native sequence of Gs, in which only the C-terminal 13 amino acids are varied, it is difficult to measure their expression and localization independent of endogenous Gs. As implemented here, the GsX assay, therefore, does not yield absolute quantification of GPCR/G-protein signaling, but rather only relative activities that must be interpreted with reference to an internal control. This is particularly important when inferring selectivity, given the limitations present in directly measuring GsX expression and localization. With an appropriate internal control (in this case, rod opsin), the GsX assay is an attractive tool for comparing relative G-protein selectivity in living cells, as a complement to other techniques [13, 23].

The GsX assay is applicable to any GPCR that has low intrinsic Gs coupling activity and it should not be used with Gs-coupled GPCRs. It is necessary to stringently test any GPCRs of interest for native Gs activity as a preliminary control experiment, as we have done here. This is a limitation, but one that nevertheless affords substantial scope for utility. GPCRs that do not activate Gs comprise 171/253 of human GPCRs with annotated G-protein coupling in the IUPHAR/BPS expert-curated database [54, 55]. Similarly, the TGF-β shedding assay [23] is limited to GPCRs that do not normally activate Gq/11/14, G12/13, or G15, since these are all capable of intrinsically activating Phospholipase-C and thus, TGF-β shedding. In this way, the two assays can be applied to partially overlapping sets of GPCRs, and provide complementary approaches to a similar problem.

## Conclusions

Animal opsins offer enormous potential for experimental and therapeutic manipulation of neurons and many other cells. They regulate processes including vision, heartbeat, insulin release, vasoconstriction, immune cell migration, endocrine signaling, and more. A fundamental aspect of opsin function that must be measured, and may be engineered, is their selectivity toward the range of different G proteins that are present in the target cell of interest. The GsX assay allows opsin coupling to proxies of diverse Gα subunits to be tested using a single biochemical pathway in live cells. We used this assay for the first side-by-side screen of diverse opsin candidates for a gene therapy for retinal degeneration, and found that chimeric opsins are generally less active than wild-type rod opsin. We also identified LWS cone opsin and a rod opsin/amphioxus opsin chimera as optogenetic tools with complementary selectivity for Gi and Go, respectively. This assay is suitable for high-throughput screening of diverse opsins to shed light on the evolution of GPCR/G-protein coupling specificity, and also for testing libraries of designed opsins engineered for specific G-protein coupling properties, including sensitivity, response amplitude, and G-protein selectivity.

## Methods

### Sequence data

The following GenBank accession sequences were used to construct expression vectors: human rod opsin (NM_000539.3), human LWS cone opsin (NM_020061.5), human SWS cone opsin (NM_001708), scallop opsin 2 (AB006455.1), amphioxus opsin 1 (AB050606.1), human metabotropic glutamate receptor 6 (NM_000843.3), mouse mGluR6 (NM_173372.2), mouse melanopsin (NM_013887.2), human melanopsin (NM_033282), human bradykinin receptor B2 (AY275465), human mu-opioid receptor 1 (AY521028.1), human GαsL (NM_000516.5), human Gαi1, human GαoA (AH002708), human Gαt1, human Gαq (NM_002072.4), human Gαz (NM_002073.3), human Gα12 (NM_007353), human Gα13 (NM_006572), and human Gα15 (AF493904).

### Expression vector construction

All opsin and opsin-based chimeras had the native stop codon replaced by a 27-bp sequence from bovine rod opsin that encodes the 1D4 monoclonal antibody tag (TETSQVAPA). The pcDNA3 human rod opsin, JellyOp, and Glo22F plasmids were described previously [12]. Plasmids encoding the LWS and SWS cone opsins were purchased from the DNASU plasmid repository [56] deposited by the Centre for Personalised Diagnostics. Plasmids encoding ScallOp2 (from *Patinopecten yessoensis*) [45] and AmphiOp1 (from *Branchiostoma belcheri*) [44] were generously provided by Prof. Akihisa Terakita (Osaka City University). A plasmid encoding the mouse melanopsin-mGluR6 chimera Opto-mGluR6 tagged with the 1D4 epitope [9] was kindly provided by Dr. Elena Lesca and Prof. Gebhard Schertler (Paul Scherrer Institute). pcDNA3 human BDKRB2, human MOR, and human Gαs plasmids were purchased from the cDNA Resource Center (www.cdna.org).

Opsin and opsin chimera sequences were transferred to the pcDNA3 vector by amplifying the desired coding sequence using PCR, and ligating this into the linearized pcDNA3 vector via Gibson assembly [57] using NEBuilder HiFi enzyme mix (New England Biolabs). Chimeric cDNA sequences encoding the rod opsin loop 3 and opsin-mGluR6 chimeras were synthesized by Thermo Fisher and ligated into the pcDNA3 vector using Gibson assembly. Open-reading-frame sequences for opsins and opsin chimeras used in this paper are listed in Additional file 9. All plasmids used have been deposited with Addgene.

The GsX chimeras were constructed by ligating annealed oligonucleotides to a human GαsL construct in which the C-terminus (encoding the last 13 amino acids) was replaced with a restriction enzyme site. Open-reading-frame sequences for GsX chimeras are listed in Additional file 9. Gsi, Gso, and Gst chimeras were designed to include a mutation of the adenosine diphosphate-ribosylation site, from cysteine to serine, rendering them insensitive to pertussis toxin [58–60]. All GsX constructs were expressed in a plasmid containing a CAG promotor and a β-globin 3' untranslated region based on pEM705 (a gift from Eugene Makeyev, UCL).

### Cell culture

HEK293T cells (ATCC) were incubated at 37 °C (5% CO_2_) in culture media: Dulbecco’s modified Eagle’s medium (DMEM) containing 4500 mg/L glucose, L-glutamine, sodium pyruvate, and sodium bicarbonate (Sigma) with penicillin (100 U/ml), streptomycin (100 μg/ml), and 10% fetal bovine serum (FBS).

For transfections, cells were seeded into 12-well plates in antibiotic-free culture media at a density of 250,000 cells/well and were transiently transfected after 48 hours using Lipofectamine 2000 (Thermo Fisher Scientific), according to the manufacturer’s instructions. Where appropriate, cells were co-transfected with a 1:1 ratio of GloSensor and GPCR plasmid (500 ng/well of each plasmid). For GsX assays, cells were also co-transfected with 5 ng/well of GsX chimera (100:100:1 ratio of GloSensor to GPCR to Gs chimera plasmid). We found that the 100:1 opsin:GsX ratio yielded optimum response amplitudes when tested with rod opsin and Gst.

After initial transfection, all steps were carried out under dim red light. Cells were incubated in transfection reagent at 37 °C for 4–6 hours, then resuspended in 1 ml of culture media with 10 μM 9-cis retinal (Sigma-Aldrich) and, for endogenous Gs and GsX assays, 125 ng/ml pertussis toxin (Sigma-Aldrich). Pertussis toxin was added to eliminate signaling via endogenous Gi pathways, which decreases cAMP.

### Immunocytochemistry

For immunocytochemistry, cells were transiently transfected and resuspended in DMEM with 9-cis retinal, as described above. The total volume of resuspended cells was then seeded onto poly-l-lysine coated 12 mm #1.5 coverslips. Cells were incubated overnight at 37 °C, then fixed using 4% paraformaldehyde (Thermo Fisher Scientific). Cells were washed in phosphate-buffered saline (PBS), then incubated in PBS with 2% glycine for 5 mins and washed in PBS. Cells were blocked in PBS with 5% bovine serum albumin (BSA) and 0.1% Triton X-100 for 30 mins at room temperature.

Cells were incubated in 1:500 dilution of monoclonal 1D4 rod opsin antibody (Abcam, catalog no. ab5417, lot no. GR272982-11, RRID AB_304874) in PBS with 2% BSA for 1 hour at room temperature, then washed with PBS and incubated in 1:2000 dilution of donkey anti-mouse Alexa594 secondary antibody (Molecular Probes, catalog no. A21203, lot no. 1722945, RRID AB_141633) in PBS with 2% BSA for 1 hour at room temperature. The secondary antibody was then removed and cells were washed and incubated in PBS with DAPI (250 ng/ml) and 0.1% Tween-20 for one minute, then washed and mounted using ProLong-Gold Anti-Fade reagent (Thermo Fisher Scientific).

Images were acquired using an Olympus BX51 upright microscope using a 20× UPlan FLN objective with excitation at 350 and 560 nm, and emission at 460 and 645 nm for DAPI and red fluorescence, respectively. Images were collected using a CoolSnap HQ camera (Photometrics) through MetaVue software (Molecular Devices) and analyzed using FIJI ImageJ [61, 62]. Ten randomly selected fields were imaged per condition. Relative levels of fluorescence intensity were quantified by measuring the integrated intensity of each field above a threshold (200), then normalizing this to the average integrated intensity of the negative control (no opsin).

### Live-cell cAMP assays

For second messenger assays, cells were transiently transfected and resuspended in 9-cis retinal (and pertussis toxin, if required) as described above. Under dim red light, 100 μl of cell suspension was then added to each well of a solid white 96-well plate (Greiner) and incubated at 37 °C overnight. Then, 1–2 hours before beginning the second messenger assays, cells were incubated at room temperature in L-15 media (without phenol-red) containing L-glutamine (Gibco), 1% FBS, penicillin (100 U/ml), and streptomycin (100 μg/ml), with 10 μM 9-cis retinal and 2 mM beetle luciferin potassium salt (Promega) reconstituted in 10 mM HEPES pH 6.9.

Glo22F was used as a bioluminescent reporter of cAMP. GPCR coupling to endogenous Gs and GsX chimeras signaling pathways was measured as the maximum cAMP post-stimulus, while stimulation of Gi pathway was measured as minimum cAMP post-stimulus. For endogenous Gi assays, forskolin (Sigma-Aldrich) was added to the cells (final concentration 2 μM) approximately 30 mins before the start of the assay [12, 27].

Luminescence was measured using a FluoStar Optima microplate reader (BMG Labtech). Raw luminescence units were recorded from above a cell using the top optic and 3 mm lens (Gain adjustment set to 3600) for 1 s, every 60 or 75 s for opsin assays, and every 2.2 mins for BKB2R and MOR assays. Baseline luminescence was recorded for five cycles, then recording was paused and the plate ejected. Cells were exposed to the stimulus then returned to the reader, and recording resumed for a minimum of ten cycles or until the response peak was observed.

For opsin experiments, cells were stimulated with a single 470 nm light flash using a custom-built LED array. Each well was exposed to one of eight different intensities over a 5-log range (from 4 × 10^12^ to 10^16^ photons). One well from each condition was left unexposed as a dark control. To monitor GPCR agonist responses, cells transfected with BDKRB2 and MOR were treated with BK (Tocris) or EM (Sigma-Aldrich), respectively. Each well was exposed to one of seven different agonist concentrations over a 7-log range (from 1 mM to 0.1 nM). One well from each condition was left untreated as a negative control. Data were collected from three or four biological replicates for each condition.

### Data processing and statistics

For second messenger assays, raw GloSensor cAMP luminescence data was first normalized by dividing by the baseline signal at the pre-stimulus timepoint. This is reported as the normalized luminescence in time-course graphs (Figs. 3b, 5c and Additional file 2: Figure S1a). The response to the stimulus was calculated for each trial as follows, and labeled the normalized response in dose–response and irradiance–response curves. For endogenous Gs and GsX assays (Figs. 1, 3, and 4), the maximum fold-increase in the GloSensor cAMP signal was calculated for each intensity of light or concentration of agonist tested. For endogenous Gi assays (Fig. 4 and Additional file 3: Figure S3), the minimum fold-decrease was calculated. ScallOp2 and AmphiOp1 were analyzed using the maximum deviation from baseline (Fig. 5).

For MOR and BDKRB2, we observed an approximately twofold systematic variation from day to day in response amplitudes across the entire set of GsX. To correct for this variation, we performed the following batch-wise normalization: the response amplitude from each replicate was divided by the average response amplitude for all replicates in that batch of data, then multiplied by the average response amplitude across all batches.

For each experiment, the data were subjected to a model comparison (F-test) in which we tested whether the data were better fit by a flat line, representing the null hypothesis of no stimulus response, or a sigmoid dose–response curve, representing the alternative hypothesis of stimulus-responsive GPCR activity with an α threshold of 0.05. The dose–response model was a three-parameter sigmoid function: *y* = *a* + *b*/(1 + 10^(*c* - *x*)^), where *a* is the baseline, *b* is the response amplitude, and *c* is logEC50 fitted by a nonlinear regression. All curve-fitting and statistical analysis were done using Graphpad Prism7 software. For those opsins that passed this test, we compared two figures of merit from the dose–response curve: response amplitude and logEC50. The results of the model comparison test and the best-fitting parameters for the dose–response curve from pooled data are shown in Additional files 1 and 7.

In general, the above analysis was performed on average pooled data from all replicates. For Fig. 3d,e, Additional file 8: Figure S6a, and Figure S6b, curves were fit to each individual replicate and compared to a flat-line model as above, and derived parameters were compared by one-way ANOVA either between opsins or between G proteins. The best-fitting parameters from individual trials are listed in Additional file 10a. For Fig. 3d and Additional file 8: Figure S6a, best-fitting response amplitude and logEC50 derived from individual trials were compared between opsins, using rod opsin as a reference, with α = 0.0033, reflecting the Bonferroni correction for multiple comparisons (initial α = 0.05, 15 comparisons in total). In Additional file 8: Figure S6b, best-fitting response amplitudes with Gsi, Gso, and Gst were compared within each opsin, for a total of 18 comparisons within this family (α = 0.0027). In Fig. 3e, we first calculated the average response amplitude for rod opsin with Gsi, Gso, and Gst, then divided the Gsi, Gso, and Gst response amplitudes for the other five opsins by the respective rod opsin average, yielding a relative response amplitude expressed as a percentage. These relative response amplitudes were then compared within each opsin, for a total of 15 comparisons (α = 0.0033). ANOVA was performed in Prism7. The details of these analyses are listed in Additional file 10b–e. Note that the *p* values listed are uncorrected, we implemented the Bonferroni correction by dividing alpha (0.05) by the number of comparisons, which is equivalent to multiplying the *p* values.

## Additional files

Additional file 1: Curve-fitting results for pooled dose–response data shown in Fig. 1d. For each GPCR/G protein combination, pooled dose–response data were analyzed by F-test comparing a three-parameter sigmoid model (test hypothesis) to a flat-line model (null hypothesis), with significance threshold *p* < 0.05. Included in this additional file are the F-statistic, degrees of freedom, and corresponding *p* value for each test. For data sets that met the significance criteria, we report the *R*-square value for the best-fitting dose–response curve and its three defining parameters: bottom, top (response amplitude), and logEC50 (sensitivity), as well as the 95% confidence intervals for those values. (XLSX 14 kb)

Additional file 2: Figure S1. Pertussis toxin, GsX point mutations, and comparison to JellyOp. **a–c** HEK293T cells transfected with Glo22F and rod opsin were treated with or without pertussis toxin (PTX) as indicated. Forskolin was added to elevate cAMP after 5 mins, and cells were flashed with light at varying intensities at 33 mins. The signal for each trial was normalized to pre-flash and the minimum cAMP post-flash was recorded for each trial. **a**,**b** Time courses of the GloSensor cAMP signal (average of three trials +/- SEM) for dark controls and 10^15.1^ photons/mm^2^ flash conditions. **a** Raw luminescence and **b** normalized to the final point prior to flash. Average responses +/-SEM for all light levels tested are shown in (**c**), with the best-fitting curve for –PTX. **d** HEK293T cells transfected with Glo22F, rod opsin, and Gso, Gsi, or Gst bearing the native Cys residue in the C-terminal -4 position were not treated with PTX. Cells were stimulated with light and the responses analyzed as in Fig. 3. Best-fitting maximum response amplitudes are graphed alongside the response amplitudes for rod opsin tested with PTX-insensitive Gso, Gsi, or Gst and treated with PTX (data reproduced from Fig. 3d). The wild-type and Ser point mutants for each GsX were compared by ANOVA. Uncorrected *p* values are shown and no differences were statistically significant. **e** HEK293T cells were transfected with Glo22F and either JellyOp or rod opsin, with or without exogenous G protein as indicated, and treated with PTX. Cells were stimulated with light and responses analyzed as in Fig. 3. The graph shows mean responses (*n* = 3, +/-SEM) at each light intensity. Lines are best-fitting sigmoid curves. Error bars smaller than symbols are not shown. (PDF 441 kb)

Additional file 3: Figure S2. Bradykinin, endomorphin-1, and light controls without GPCRs. HEK293T cells were transfected with Glo22F only (-), wild-type Gs (s), or GsX chimeras and treated with **a** 100 μM bradykinin (BK), **b** 100 μM endomorphin-1 (EM), or **c** 470 nm light (10^14.1^ photons/mm^2^) to test whether cells exhibited a cAMP response to any of these stimuli in the absence of transfected receptors or opsin. The signal from each trial was normalized to the pre-stimulus baseline and the maximum post-stimulus level was recorded (within 20 mins for BKB2R and MOR, or 10 mins for rod opsin). Individual responses, mean, and SEM of three technical replicates are shown. (PDF 408 kb)

Additional file 4: Figure S3. Effects of opsin and Gso, Gsi, or Gst transfection on basal GloSensor cAMP levels. Baseline cAMP for –opsin and + opsin conditions, -/+ Gsi, Gso, or Gst, normalized to mean of –opsin, -GsX condition. Box and whisker plots show mean, 25th percentile, 75th percentile, and range, *n* ≥ 23, for each condition. The baseline cAMP reporter signal is highly variable, likely reflecting variation in transfection efficiency and cell number as well as systematic effects of different GsX proteins and opsins. Nevertheless, three trends emerged. (1) Transfecting opsins alone did not increase basal cAMP. (2) Transfecting Gsi, Gso, or Gst elevated baseline cAMP in the absence of opsin, presumably through the background activity of endogenous Gi/o/t-coupled GPCRs. (3) Transfecting opsins in combination with Gsi, Gso, or Gst elevated basal cAMP above the level achieved by GsX transfection. This is expected, as opsins are known to have non-zero G-protein activation in the dark. (PDF 408 kb)

Additional file 5: Figure S4. Testing opsin coupling to endogenous Gs. To test potential opsin coupling to endogenous Gs, HEK293T cells were transfected with Glo22F and opsins treated with pertussis toxin, and exposed to 470 nm light for *n* = 3 replicates. The signal for each trial was normalized to pre-flash. The maximum cAMP post-flash was recorded for each trial. Graphs show mean cAMP response +/- SEM at varying irradiance for each opsin. RL3Am and RL3m6L2 exhibited responses that satisfied our statistical criteria (fitted response curves are shown). Error bars smaller than symbols are not shown. (PDF 454 kb)

Additional file 6: Figure S5. Testing opsin coupling to endogenous Gi. To test opsin coupling to endogenous Gi, HEK293T cells were transfected with Glo22F and opsins treated with forskolin (2 μM), and exposed to 470 nm light for *n* = 3 replicates. The signal for each trial was normalized to pre-flash. The minimum cAMP post-flash was recorded for each trial. Graphs show mean cAMP response +/- SEM at varying irradiance for each opsin. Fits show sigmoidal dose–response curves. AmphiOp1 did not exhibit a statistically significant light response. Gi assay results for mML23Cm6, hML23Cm6, and RL23Cm6 are shown in Fig. 4 and results for ScallOp2 are shown in Fig. 5. Error bars smaller than symbols are not shown. (PDF 506 kb)

Additional file 7: Curve-fitting results for pooled dose–response data shown in Fig. 3c. For each opsin/G-protein combination, pooled irradiance–response data were analyzed with an F-test comparing a three-parameter sigmoid model (test hypothesis) to a flat-line model (null hypothesis), with significance threshold *p* < 0.05. Included in this additional file are the F-statistic, degrees of freedom, and corresponding *p* value for each test. For data sets that met the significance criteria, we report the *R*-square value for the best-fitting irradiance–response curve and its three defining parameters: bottom, top (response amplitude), and logEC50 (sensitivity), as well as the 95% confidence intervals for those values. (XLSX 16 kb)

Additional file 8: Figure S6. Opsin sensitivity. Selectivity comparison without normalization to rod opsin. **a** Log10(EC50) values from best-fitting sigmoid curves in Fig. 3d are plotted (see also Additional file 10). EC50 values were compared within each GsX by ANOVA. α = 0.0033 (reflecting Bonferroni correction, 15 comparisons). Asterisks (*) indicate significant differences. **b** Response amplitudes as shown in Fig. 3d were analyzed by ANOVA, comparing the responses of each Gsi, Gso, and Gst within each opsin, without prior normalization to rod opsin. For this comparison, α = 0.0027 (reflecting Bonferroni correction, 18 comparisons). Asterisks (*) indicate significant differences. (PDF 408 kb)

Additional file 9: Open reading frame DNA sequences for the opsin and GsX proteins used in this study. (docx 23 kb)

Additional file 10: Curve-fitting results for individual dose–response data in Fig. 3 and results of ANOVAs shown in Fig. 3 and Additional file 8: Figure S6. For RHO, LWS cone opsin, SWS cone opsin, RL3Sc, RL3Am, and RL3m6L2, the irradiance–response data from each individual trial was analyzed with an F-test comparing a three-parameter sigmoid model (test hypothesis) to a flat-line model (null hypothesis), with significance threshold *p* < 0.05 (same analysis as with the pooled data). **a** Details of curve-fitting to individual irradiance–response data, including the F-statistic, degrees of freedom, and corresponding *p* value for each test, and the best-fitting parameters that define the irradiance–response curve: bottom, top (response amplitude), and logEC50 (sensitivity). **b** Relevant details of the ANOVA tests in Fig. 3d. **c** Relevant details of the ANOVA tests in Fig. 3e. **d** Relevant details of ANOVA test in Additional file 8: Figure S6a. **e** Relevant details of ANOVA test in Additional file 8: Figure S6b. (XLSX 24 kb)

Additional file 11: Raw and analyzed data for all multi-well luminescent recordings used to generate Figs. 1, 2, 3, 4, and 5 and Additional file 2: Figure S1, Additional file 3: Figure S2, Additional file 4: Figure S3, Additional file 5: Figure S4, Additional file 6: Figure S5, and Additional file 8: Figure S6. (XLSX 1795 kb)
