# Supplementary figures and images for: A live cell assay of GPCR coupling allows identification of optogenetic tools for controlling Go and Gi signaling
Source: BMC Biol. 2018 Jan 16;16:10. doi: 10.1186/s12915-017-0475-2 (PMC5771134; doi:10.1186/s12915-017-0475-2)

Figure S1

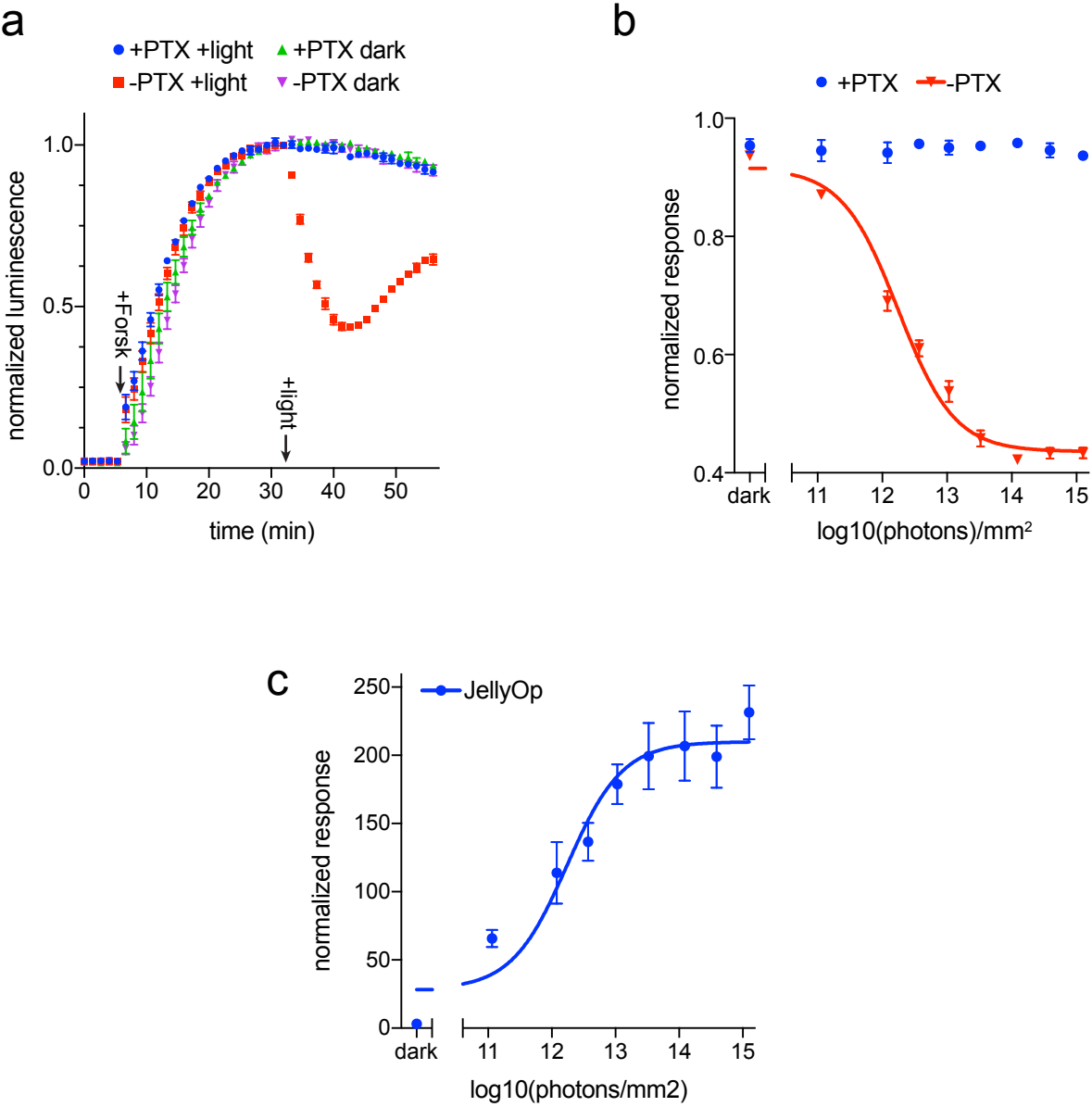

Supplement: Supplementary file 2 — Pertussis toxin, GsX point mutations, and comparison to JellyOp. a–c HEK293T cells transfected with Glo22F and rod opsin were treated with or without pertussis toxin (PTX) as indicated. Forskolin was added to elevate cAMP after 5 mins, and cells were flashed with light at varying intensities at 33 mins. The signal for each trial was normalized to pre-flash and the minimum cAMP post-flash was recorded for each trial. a,b Time courses of the GloSensor cAMP signal (average of three trials +/- SEM) for dark controls and 1015.1 photons/mm2 flash conditions. a Raw luminescence and b normalized to the final point prior to flash. Average responses +/-SEM for all light levels tested are shown in (c), with the best-fitting curve for –PTX. d HEK293T cells transfected with Glo22F, rod opsin, and Gso, Gsi, or Gst bearing the native Cys residue in the C-terminal -4 position were not treated with PTX. Cells were stimulated with light and the responses analyzed as in Fig. 3. Best-fitting maximum response amplitudes are graphed alongside the response amplitudes for rod opsin tested with PTX-insensitive Gso, Gsi, or Gst and treated with PTX (data reproduced from Fig. 3d). The wild-type and Ser point mutants for each GsX were compared by ANOVA. Uncorrected p values are shown and no differences were statistically significant. e HEK293T cells were transfected with Glo22F and either JellyOp or rod opsin, with or without exogenous G protein as indicated, and treated with PTX. Cells were stimulated with light and responses analyzed as in Fig. 3. The graph shows mean responses (n = 3, +/-SEM) at each light intensity. Lines are best-fitting sigmoid curves. Error bars smaller than symbols are not shown. (PDF 441 kb) [file 12915_2017_475_MOESM2_ESM.pdf]

Figure S2

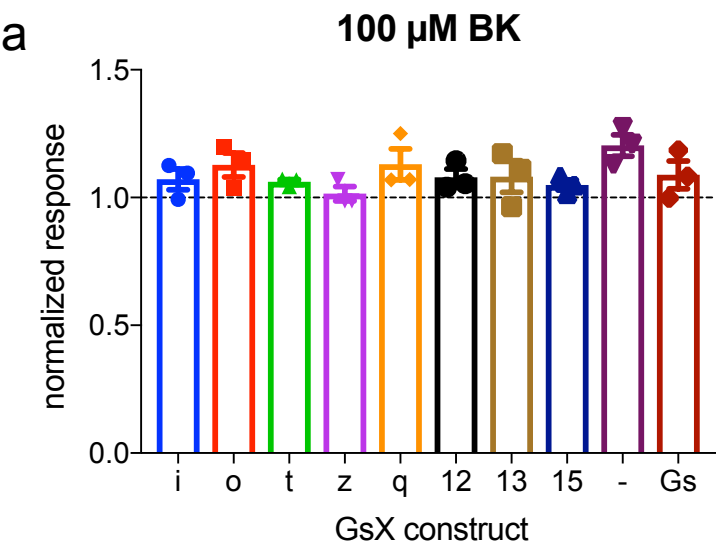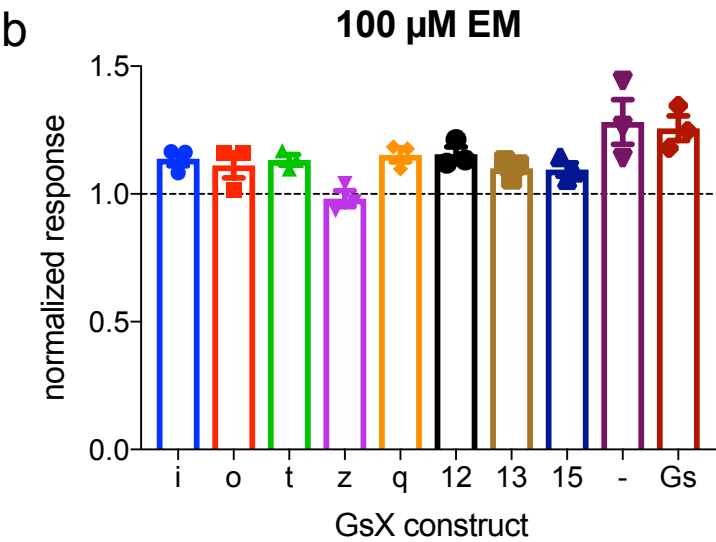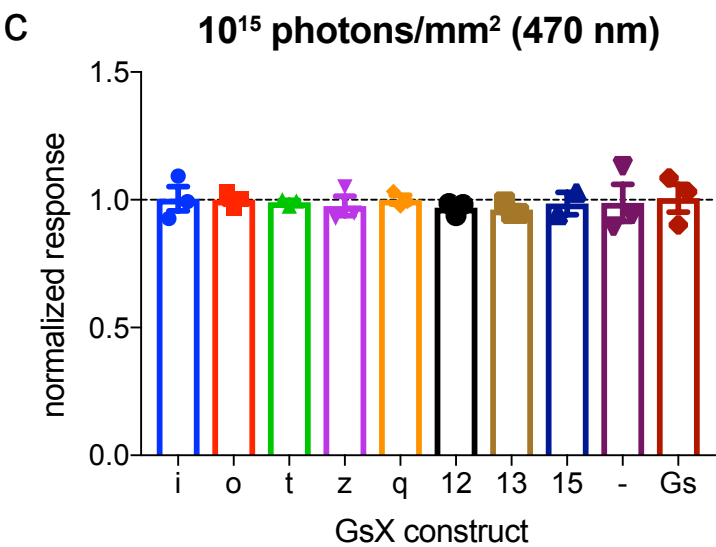

Supplement: Supplementary file 3 — Bradykinin, endomorphin-1, and light controls without GPCRs. HEK293T cells were transfected with Glo22F only (-), wild-type Gs (s), or GsX chimeras and treated with a 100 μM bradykinin (BK), b 100 μM endomorphin-1 (EM), or c 470 nm light (1014.1 photons/mm2) to test whether cells exhibited a cAMP response to any of these stimuli in the absence of transfected receptors or opsin. The signal from each trial was normalized to the pre-stimulus baseline and the maximum post-stimulus level was recorded (within 20 mins for BKB2R and MOR, or 10 mins for rod opsin). Individual responses, mean, and SEM of three technical replicates are shown. (PDF 408 kb) [file 12915_2017_475_MOESM3_ESM.pdf]

Figure S3

a

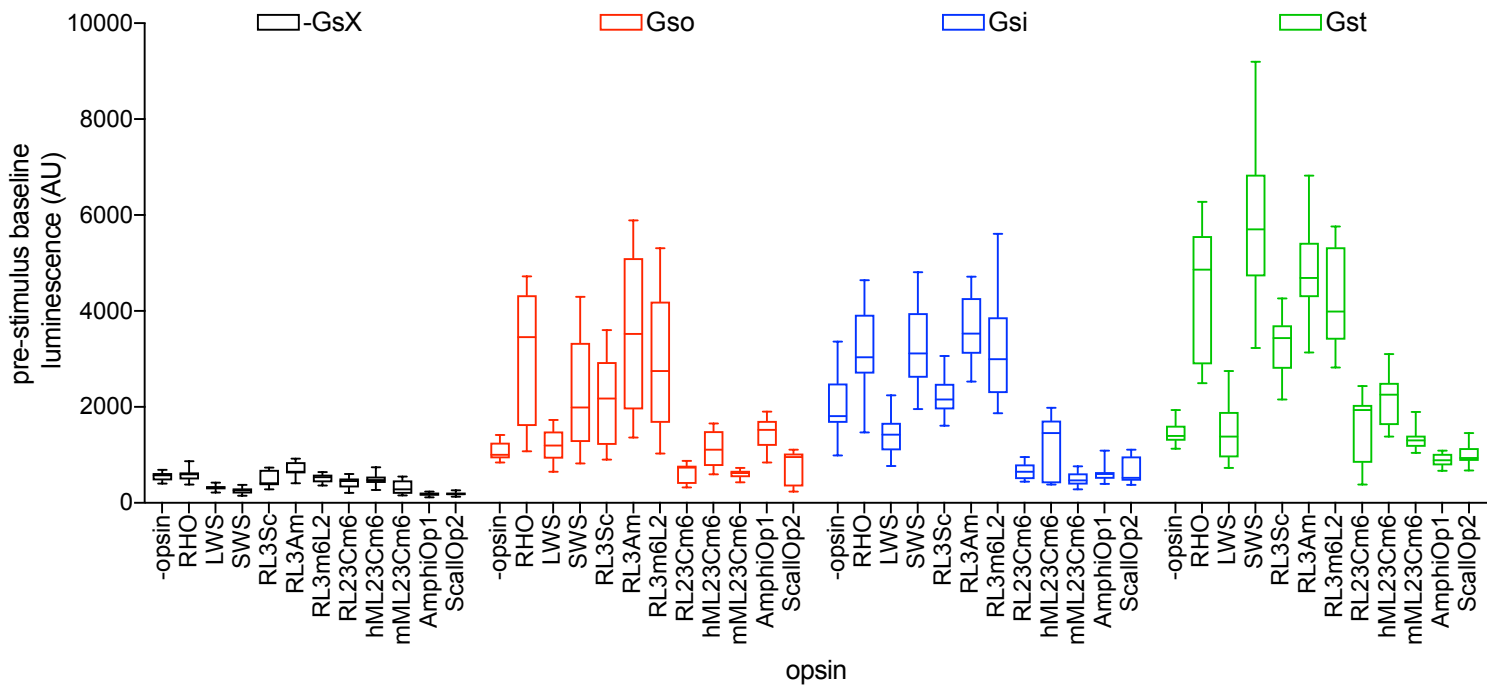

Supplement: Supplementary file 4 — Effects of opsin and Gso, Gsi, or Gst transfection on basal GloSensor cAMP levels. Baseline cAMP for –opsin and + opsin conditions, -/+ Gsi, Gso, or Gst, normalized to mean of –opsin, -GsX condition. Box and whisker plots show mean, 25th percentile, 75th percentile, and range, n ≥ 23, for each condition. The baseline cAMP reporter signal is highly variable, likely reflecting variation in transfection efficiency and cell number as well as systematic effects of different GsX proteins and opsins. Nevertheless, three trends emerged. (1) Transfecting opsins alone did not increase basal cAMP. (2) Transfecting Gsi, Gso, or Gst elevated baseline cAMP in the absence of opsin, presumably through the background activity of endogenous Gi/o/t-coupled GPCRs. (3) Transfecting opsins in combination with Gsi, Gso, or Gst elevated basal cAMP above the level achieved by GsX transfection. This is expected, as opsins are known to have non-zero G-protein activation in the dark. (PDF 408 kb) [file 12915_2017_475_MOESM4_ESM.pdf]

Figure S4

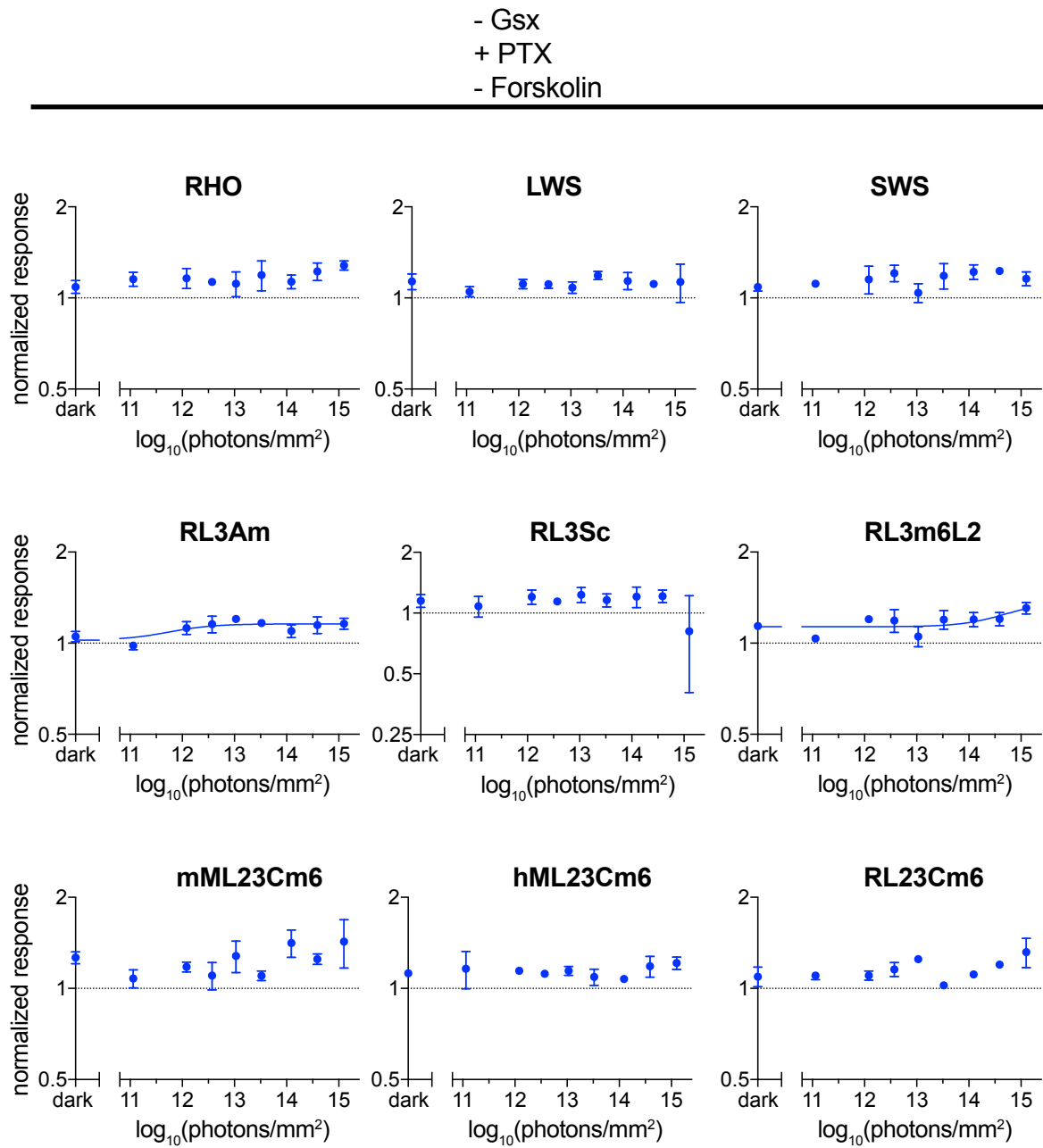

Supplement: Supplementary file 5 — Testing opsin coupling to endogenous Gs. To test potential opsin coupling to endogenous Gs, HEK293T cells were transfected with Glo22F and opsins treated with pertussis toxin, and exposed to 470 nm light for n = 3 replicates. The signal for each trial was normalized to pre-flash. The maximum cAMP post-flash was recorded for each trial. Graphs show mean cAMP response +/- SEM at varying irradiance for each opsin. RL3Am and RL3m6L2 exhibited responses that satisfied our statistical criteria (fitted response curves are shown). Error bars smaller than symbols are not shown. (PDF 454 kb) [file 12915_2017_475_MOESM5_ESM.pdf]

Figure S5

- Gsx  
- PTX  
+ Forskolin

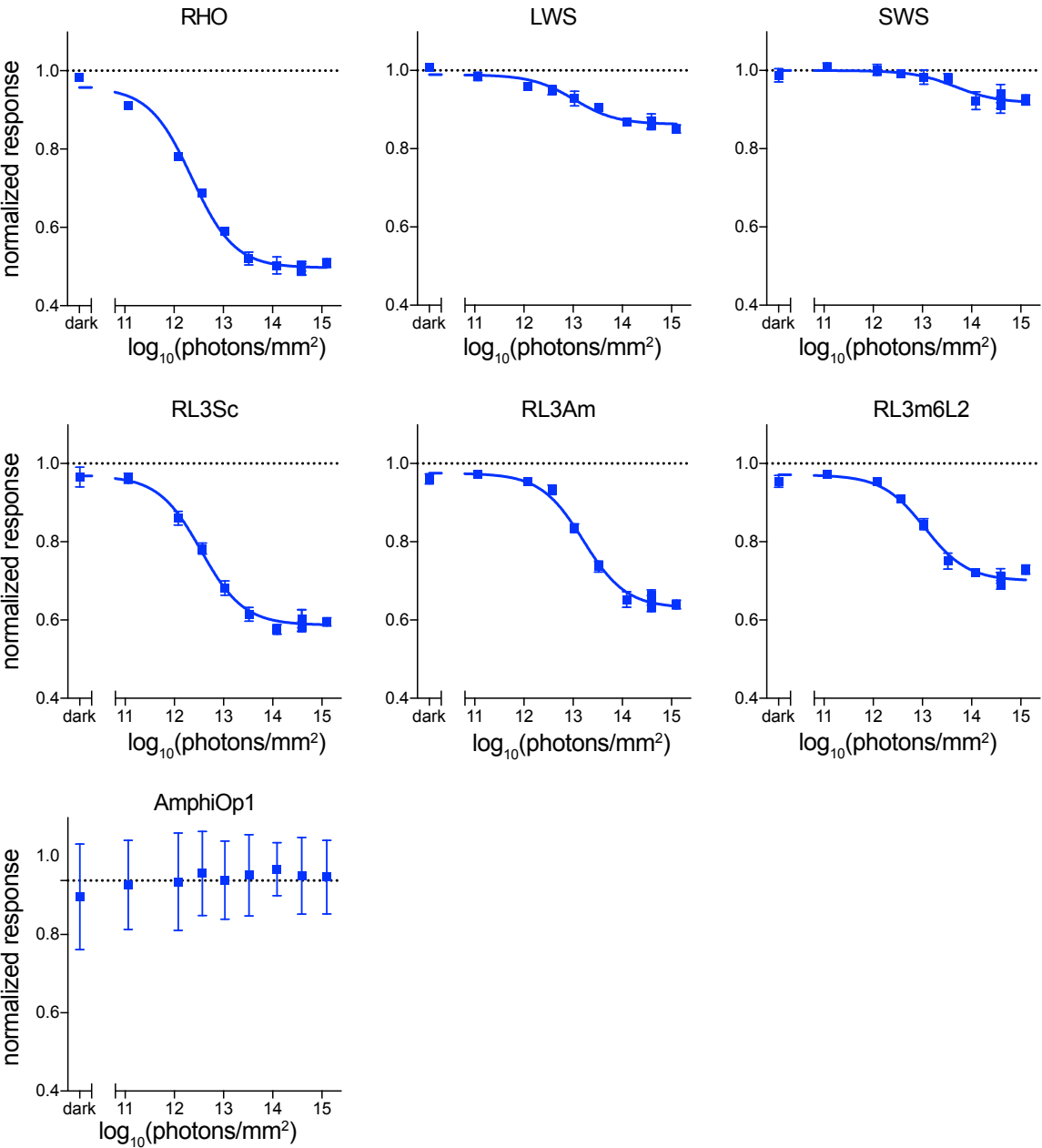

Supplement: Supplementary file 6 — Testing opsin coupling to endogenous Gi. To test opsin coupling to endogenous Gi, HEK293T cells were transfected with Glo22F and opsins treated with forskolin (2 μM), and exposed to 470 nm light for n = 3 replicates. The signal for each trial was normalized to pre-flash. The minimum cAMP post-flash was recorded for each trial. Graphs show mean cAMP response +/- SEM at varying irradiance for each opsin. Fits show sigmoidal dose–response curves. AmphiOp1 did not exhibit a statistically significant light response. Gi assay results for mML23Cm6, hML23Cm6, and RL23Cm6 are shown in Fig. 4 and results for ScallOp2 are shown in Fig. 5. Error bars smaller than symbols are not shown. (PDF 506 kb) [file 12915_2017_475_MOESM6_ESM.pdf]

Figure S6

a

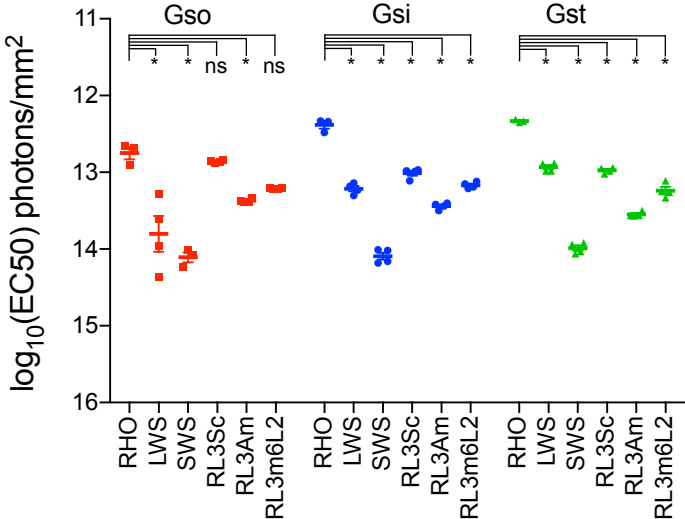

b

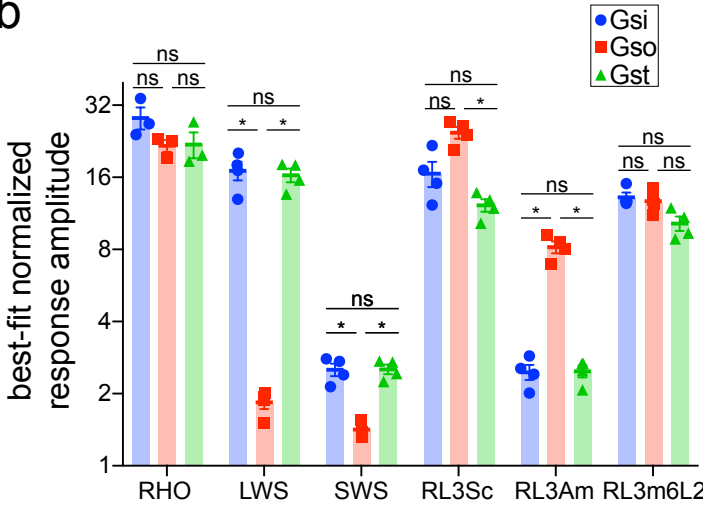

Supplement: Supplementary file 8 — Opsin sensitivity. Selectivity comparison without normalization to rod opsin. a Log10(EC50) values from best-fitting sigmoid curves in Fig. 3d are plotted (see also Additional file 10). EC50 values were compared within each GsX by ANOVA. α = 0.0033 (reflecting Bonferroni correction, 15 comparisons). Asterisks (*) indicate significant differences. b Response amplitudes as shown in Fig. 3d were analyzed by ANOVA, comparing the responses of each Gsi, Gso, and Gst within each opsin, without prior normalization to rod opsin. For this comparison, α = 0.0027 (reflecting Bonferroni correction, 18 comparisons). Asterisks (*) indicate significant differences. (PDF 408 kb) [file 12915_2017_475_MOESM8_ESM.pdf]
