# Supplementary material for: A live cell assay of GPCR coupling allows identification of optogenetic tools for controlling Go and Gi signaling
Source: BMC Biol. 2018 Jan 16;16:10. doi: 10.1186/s12915-017-0475-2 (PMC5771134; doi:10.1186/s12915-017-0475-2)
Supplement: Supplementary file 9 — Open reading frame DNA sequences for the opsin and GsX proteins used in this study. (docx 23 kb) [file 12915_2017_475_MOESM9_ESM.docx]

**Additional file 9: open reading frame sequences**

**Chimeric regions are shown in BOLD CAPS**

1D4 epitope sequence is underlined

**>RHO**

ATGaatggcacagaaggccctaacttctacgtgcccttctccaatgcgacgggtgtggtacgcagccccttcgagtacccacagtactacctggctgagccatggcagttctccatgctggccgcctacatgtttctgctgatcgtcctaggcttccccataaatttcctcacgctctacgtcaccgtccagcacaagaagctgcgcacgcctctcaactacatcctgctcaacctagccgtggcagatctcttcatggttctaggtggcttcaccagcaccctctacacctctctgcatggatacttcgtcttcgggcccacaggatgcaatttggagggcttctttgccaccctgggcggtgaaattgccctgtggtccttggtggtcctggccatcgagcggtacgtggtggtgtgtaagcccatgagcaacttccgcttcggggagaaccatgccatcatgggcgttgccttcacgtgggtcatggcgctggcctgcgccgcacccccactcgccggctggtccaggtacatccccgagggcctgcagtgctcgtgtggaatcgactactacacgctcaagccggaggtcaacaacgagtcttttgtcatctacatgttcgtggtccacttcaccatccccatgattatcatctttttctgctatgggcagctcgtcttcaccgtcaaggaggccgctgcccagcagcaggagtcagccaccacacagaaggcagagaaggaggtcacccgcatggtcatcatcatggtcatcgctttcctcatatgctgggtgccctacgccagcgtggcattctacatcttcacccaccagggctccaacttcggtcccatcttcatgaccatcccagcgttctttgccaagagcgctgccatctacaaccctgtcatctatatcatgatgaacaagcagttccggaactgcatgctcaccaccatctgctgcggcaagaacccactgggtgacgatgaggcctctgctaccgtgtccaagacggagacgagccaggtggccccggccTAA

**>LWS**

ATGgcccagcagtggagcctccaaaggctcgcaggccgccatccgcaggacagctatgaggacagcacccagtccagcatcttcacctacaccaacagcaactccaccagaggccccttcgaaggcccgaattaccacatcgctcccagatgggtgtaccacctcaccagtgtctggatgatctttgtggtcactgcatccgtcttcacaaatgggcttgtgctggcggccaccatgaagttcaagaagctgcgccacccgctgaactggatcctggtgaacctggcggtcgctgacctagcagagaccgtcatcgccagcactatcagcattgtgaaccaggtctctggctacttcgtgctgggccaccctatgtgtgtcctggagggctacaccgtctccctgtgtgggatcacaggtctctggtctctggccatcatttcctgggagaggtggctggtggtgtgcaagccctttggcaatgtgagatttgatgccaagctggccatcgtgggcattgccttctcctggatctggtctgctgtgtggacagccccgcccatctttggttggagcaggtactggccccacggcctgaagacttcatgcggcccagacgtgttcagcggcagctcgtaccccggggtgcagtcttacatgattgtcctcatggtcacctgctgcatcatcccactcgctatcatcatgctctgctacctccaagtgtggctggccatccgagcggtggcaaagcagcagaaagagtctgaatccacccagaaggcagagaaggaagtgacgcgcatggtggtggtgatgatctttgcgtactgcgtctgctggggaccctacaccttcttcgcatgctttgctgctgccaaccctggttacgccttccaccctttgatggctgccctgccggcctactttgccaaaagtgccactatctacaaccccgttatctatgtctttatgaaccggcagtttcgaaactgcatcttgcagcttttcgggaagaaggttgacgatggctctgaactctccagcgcctccaaaacggaggtctcatctgtgtcctcggtatcgcctgcaacggagacgagccaggtggccccggccTAA

**>SWS**

ATGagaaaaatgtcggaggaagagttttatctgttcaaaaatatctcttcagtggggccgtgggatgggcctcagtaccacattgcccctgtctgggccttctacctccaggcagctttcatgggcactgtcttccttatagggttcccactcaatgccatggtgctggtggccacactgcgctacaaaaagttgcggcagcccctcaactacattctggtcaacgtgtccttcggaggcttcctcctctgcatcttctctgtcttccctgtcttcgtcgccagctgtaacggatacttcgtcttcggtcgccatgtttgtgctttggagggcttcctgggcactgtagcaggtctggttacaggatggtcactggccttcctggcctttgagcgctacattgtcatctgtaagcccttcggcaacttccgcttcagctccaagcatgcactgacggtggtcctggctacctggaccattggtattggcgtctccatcccacccttctttggctggagccggttcatccctgagggcctgcagtgttcctgtggccctgactggtacaccgtgggcaccaaataccgcagcgagtcctatacgtggttcctcttcatcttctgcttcattgtgcctctctccctcatctgcttctcctacactcagctgctgagggccctgaaagctgttgcagctcagcagcaggagtcagctacgacccagaaggctgaacgggaggtgagccgcatggtggttgtgatggtaggatccttctgtgtctgctacgtgccctacgcggccttcgccatgtacatggtcaacaaccgtaaccatgggctggacttacggcttgtcaccattccttcattcttctccaagagtgcttgcatctacaatcccatcatctactgcttcatgaataagcagttccaagcttgcatcatgaagatggtgtgtgggaaggccatgacagatgaatccgacacatgcagctcccagaaaacagaagtttctactgtctcgtctacccaagttggccccaacacggagacgagccaggtggccccggccTAA

**>ScallOp2**

ATGccctttccactgaatcggacagacacagccctggtcattagcccaagcgagtttagaatcatcgggatctttattagtatttgttgcatcattggcgtgctggggaacctgctgatcattatcgtcttcgccaagcggagatccgtgaggcgccccattaacttctttgtgctgaatctggccgtctctgacctgatcgtggctctgctgggctatcctatgacagccgcttccgcattctctaacaggtggatcttcgataacatcggatgcaagatctacgccttcctgtgttttaatagcggcgtgatctccattatgacccacgcagccctgagcttctgcagatacatcatcatctgtcagtacggatacaggaagaaaattactcagaccacagtgctgcgcaccctgttcagtatctggtcattcgccatgttttggaccctgtcccccctgtttggctggagctcctacgtgatcgaggtggtccctgtgtcatgcagcgtcaactggtacggccatgggctgggagacgtgtcttataccatcagtgtgattgtcgccgtgtacgtcttccctctgtcaatcattgtctttagctatgggatgattctgcaggagaaagtgtgtaaagactcccggaagaatggaatcagagctcagcagaggtacaccccacgcttcatccaggatattgaacagagagtgacattcatctcttttctgatgatggctgccttcatggtggcttggactccctacgcaattatgtccgctctggcaatcgggtctttcaacgtggagaatagttttgccgctctgcctaccctgttcgccaaagctagctgtgcttacaaccccttcatctacgccttcaccaacgccaattttcgcgacactgtggtcgaaatcatggcaccttggactacccgacgagtgggcgtcagtaccctgccatggcctcaggtcacatactatccaagaaggcgcactagcgccgtgaacacaactgatatcgagttcccagacgataacatctttattgtgaactctagtgtcaatggccccacagtcaagcgagaaaaaattgtgcagcgcaacccaatcaatgtgcggctggggatcaagattgagccccgagactcacgggcagccactgaaaataccttcacagccgattttagcgtgatcgagactagtcaggtggctcctgctTAA

**>AmphiOp1**

ATGaacgcctctcccagttcctggctgtccagcggggagttctttacggacagcccggagaactccagcgagtggccctggacggacggcccaaccgacacgacatggcgccatcaccagtctgtagactccgtgagctatgaaggctacctcgcaagtgctatctacattaccctcacagggttgattgccttctttgggaacgtcattaccatcacggtgttcctgacggaaaaggagttccgtaaaaaacagcagaacggttttgttctgaatctggccatagctgacttgagcgtctgcgtcttcgcatatccctcatctgctatagcagggtacgcaggaagatgggtgctgggagacgttggctgtacaatctatggattcctctgcttcacctttgccctggtgagcatggtcaccctgtgtgtcatcagcatctaccgctacatcctcatctgtaaaccacaatatgctcatctgctgacccatcgcaggaccgtgtatgtgatcatcgggacctggctgtacgctctggtgttcacggtccctccgctagtcggcgtcaagcgctacacttacgaaccaatgcagatcacctgctccttggactggaacgtgcagcaccccggagagaaggcctacatcgcggcggttcttgtcatcgtctatgtcctgcaggtcctgatcatgtgtttctgctacttcaacatcatcttcaagtcggctaacctcaagtttgcggctctggccagtgagaaaacaaagatggctgcaaagaaagacacctggaagacctcagtgatgtgcctgaccatggtggtgtccttcctgatcgcctggaccccgtatgccgtgtcctctacctgggacatcctgtctgctgaagatctgcccatcatcgccaccatcctgccgagcttgttcgccaagtcgtcctgcatgatgaaccccatcatctacgcctgctgtaacaccaagttccgccaggcggcagtcaagtccttccgcaagctGtgcggtatgtgcaaacagaaggttcccttgtctacaccacaagtggtgctggcaatgcagagaaacacggagttcactagtactgttgaacccaccggtcaggccttccccatgagggttcttccctctattagcgctacgcatacggctttggagactagtcaggtggctcctgctTAA

**>RL3Sc**

ATGaatggcacagaaggccctaacttctacgtgcccttctccaatgcgacgggtgtggtacgcagccccttcgagtacccacagtactacctggctgagccatggcagttctccatgctggccgcctacatgtttctgctgatcgtcctaggcttccccataaatttcctcacgctctacgtcaccgtccagcacaagaagctgcgcacgcctctcaactacatcctgctcaacctagccgtggcagatctcttcatggttctaggtggcttcaccagcaccctctacacctctctgcatggatacttcgtcttcgggcccacaggatgcaatttggagggcttctttgccaccctgggcggtgaaattgccctgtggtccttggtggtcctggccatcgagcggtacgtggtggtgtgtaagcccatgagcaacttccgcttcggggagaaccatgccatcatgggcgttgccttcacgtgggtcatggcgctggcctgcgccgcacccccactcgccggctggtccaggtacatccccgagggcctgcagtgctcgtgtggaatcgactactacacgctcaagccggaggtcaacaacgagtcttttgtcatctacatgttcgtggtccacttcaccatccccatgattatcatctttttctgctatgggcagctcgtcttcaccgtcaagg**tatgcaaagattcccgaaagaacggcattcgcgcccagcaaagatatacaccccgctttatacaagacattgaacaacgcgtaacattc**atggtcatcatcatggtcatcgctttcctcatatgctgggtgccctacgccagcgtggcattctacatcttcacccaccagggctccaacttcggtcccatcttcatgaccatcccagcgttctttgccaagagcgctgccatctacaaccctgtcatctatatcatgatgaacaagcagttccggaactgcatgctcaccaccatctgctgcggcaagaacccactgggtgacgatgaggcctctgctaccgtgtccaagacggagacgagccaggtggccccggctTAA

**>RL3Am**

ATGaatggcacagaaggccctaacttctacgtgcccttctccaatgcgacgggtgtggtacgcagccccttcgagtacccacagtactacctggctgagccatggcagttctccatgctggccgcctacatgtttctgctgatcgtcctaggcttccccataaatttcctcacgctctacgtcaccgtccagcacaagaagctgcgcacgcctctcaactacatcctgctcaacctagccgtggcagatctcttcatggttctaggtggcttcaccagcaccctctacacctctctgcatggatacttcgtcttcgggcccaccggctgcaacctggaaggcttctttgccacactcggcggcgagattgctctgtggtcactggtggtgctggccatcgagagatacgtggtcgtgtgcaagcccatgagcaacttcagattcggcgagaaccacgccatcatgggcgtcgcctttacatgggttatggccctggcttgtgccgctcctccacttgctggctggtccagatatatccctgagggcctgcagtgcagctgcggcatcgattactacaccctgaagcctgaagtgaacaacgagagcttcgtgatctacatgttcgtggtgcacttcacgatcccgatgatcattatattcttttgctacggccagctggtgttcaccgtgaag**tccgccaacctgaagtttgccgctctggccagcgaaaagaccaagatggccgccaagaaagacacctggaag**atggtcattattatggttatcgccttcctgatctgctgggtgccctatgccagcgtggccttctacatcttcacccaccaaggcagcaacttcggccccatctttatgacaatccccgccttcttcgccaagagcgccgccatctacaaccccgtgatctatatcatgatgaacaagcagttccgcaactgcatgctgaccaccatctgctgcggcaagaatcctctgggagatgatgaggccagcgccaccgtgtctaagaccgagacatctcaggtggcccctgccTAA

**>RL3m6L2** ATGaatggcacagaaggccctaacttctacgtgcccttctccaatgcgacgggtgtggtacgcagccccttcgagtacccacagtactacctggctgagccatggcagttctccatgctggccgcctacatgtttctgctgatcgtcctaggcttccccataaatttcctcacgctctacgtcaccgtccagcacaagaagctgcgcacgcctctcaactacatcctgctcaacctagccgtggcagatctcttcatggttctaggtggcttcaccagcaccctctacacctctctgcatggatacttcgtcttcgggcccacaggatgcaatttggagggcttctttgccaccctgggcggtgaaattgccctgtggtccttggtggtcctggccatcgagcggtacgtggtggtgtgtaagcccatgagcaacttccgcttcggggagaaccatgccatcatgggcgttgccttcacgtgggtcatggcgctggcctgcgccgcacccccactcgccggctggtccaggtacatccccgagggcctgcagtgctcgtgtggaatcgactactacacgctcaagccggaggtcaacaacgagtcttttgtcatctacatgttcgtggtccacttcaccatccccatgattatcatctttttctgctatgggcagctcgtcttcaccgtc**cgtatctaccgcatcttcgaacagggcaagcgctcggtcacaccccctcccttcatcagccccacctcacag**atggtcatcatcatggtcatcgctttcctcatatgctgggtgccctacgccagcgtggcattctacatcttcacccaccagggctccaacttcggtcccatcttcatgaccatcccagcgttctttgccaagagcgctgccatctacaaccctgtcatctatatcatgatgaacaagcagttccggaactgcatgctcaccaccatctgctgcggcaagaacccactgggtgacgatgaggcctctgctaccgtgtccaagacggagacgagccaggtggccccggctTAA

**>RL23Cm6**

ATGaacggcaccgagggccccaacttctacgtgcccttcagcaatgccaccggcgtcgtgcggagcccctttgagtacccccagtactacctggccgagccctggcagtttagcatgctggccgcctacatgttcctgctgatcgtgctgggcttccctatcaacttcctgaccctgtacgtgaccgtgcagcacaagaagctgcggacccccctgaactacatcctgctgaatctggccgtggccgacctgtttatggtgctgggcggcttcaccagcaccctgtacacaagcctgcacggctacttcgtgttcggccccaccggctgcaacctggaaggcttctttgccacactgggcggcgagatcgctctgtggtcactggtggtgctggccatc**gagcggatctaccggatcttcgagcagggcaagcggagcgtgacccccccaccttttatcagccct**accagccaggccatcatgggcgtggccttcacatgggtcatggccctggcttgtgccgcccctccactggctggctggtccagatatatccccgagggcctgcagtgcagctgcggcatcgattactacaccctgaagcccgaagtgaacaacgagagcttcgtgatctacatgtttgtggtgcacttcaccatccctatgatcatcatattcttttgctacggccagctggtgttcaccgtg**aaagccagaggcgtgcccgagacattcaacgaggcc**aagatggtcattattatggtcattgcctttctgatctgttgggtgccctacgcctccgtggccttttacatcttcacacatcaaggcagcaacttcgggcccatctttatgaccatccccgcctttttcgccaagagcgccgccatctacaaccccgtgatctatatcatgatg**caccccgagcagaacgtgcagaagcggaagagaagcctgaaagccacctccaccgtggccgctcctccaaagggcgaagatgccgaggcccacaag**accgagacatcccaggtggcccctgccTGA

>**hML23Cm6** ATGaaccctccttcggggccaagagtcccgcccagcccaacccaagagcccagctgcatggccaccccagcaccacccagctggtgggacagctcccagagcagcatctccagcctgggccggcttccatccatcagtcccacagcacctgggacttgggctgctgcctgggtccccctccccacggttgatgttccagaccatgcccactataccctgggcacagtgatcttgctggtgggactcacggggatgctgggcaacctgacggtcatctataccttttgcaggagcagaagcctccggacacctgccaacatgttcattatcaacctcgcggtcagcgacttcctcatgtccttcacccaggcccctgtcttcttcaccagtagcctctataagcagtggctctttggggagacaggctgcgagttctatgccttctgtggagctctctttggcatttcctccatgatcaccctgacggccatcgccctg**gaccgtatctaccgcatctttgagcagggcaagcgctcggtcacaccccctcccttcatcagcccc**acctcacaggtcctgctgggcgtttggctctatgccctggcctggagtctgccacccttcttcggctggagcgcctacgtgcccgaggggttgctgacatcctgctcctgggactacatgagcttcacgccggccgtgcgtgcctacaccatgcttctctgctgcttcgtgttcttcctccctctgcttatcatcatctactgctacatcttcatcttc**agggcccgtggcgtgcccgagaccttcaacgaggcc**aagatcatgctgctggtcatcctcctcttcgtgctctcctgggctccctattccgctgtggccctggtggcctttgctgggtacgcacacgtcctgacaccctacatgagctcggtgccagccgtcatcgccaaggcctctgcaatccacaaccccatcatttacgccatcacc**caccccgagcagaatgtgcagaagcgaaagcggagcctcaaggccacctccacggtggcagccccacccaagggcgaggatgcagaggcccacaag**acggagactagccaggtggccccggccTAG

**>mML23Cm6**

ATGgactctccttcaggaccaagagtcttgtctagcttaactcaggaccccagcttcacaaccagtcctgccctgcaaggcatttggaacggcactcagaacgtctccgtaagagcccagcttctctctgttagccccacgacatctgcacatcaggctgctgcctgggtccccttccccacagtcgatgtcccagaccatgctcactatacccttggcacggtgatcctgctggtgggactcacagggatgctgggcaatctgacggtcatctacaccttttgcaggaacagaggcctgcggacaccagcaaacatgttcatcatcaacctcgcagtcagcgacttcctcatgtcagtcactcaggccccggtcttctttgccagcagcctctacaagaagtggctctttggggagacaggttgcgagttctatgccttctgcggggctgtctttggcatcacttccatgatcaccctgacagccatagccatg**gaccgcatctaccgcattttcgagcaagggaagcgctctgtcacgccgccacccttcatcagcccc**acctcgcaggtcctgctaggcgtctggctttatgccctggcctggagtctgccacctttctttggttggagtgcctacgtgcccgaggggctgctgacatcctgctcctgggactacatgaccttcacaccccaggtgcgtgcctacaccatgctgctcttctgctttgtcttcttcctccccctgctcatcatcatcttctgctacatcttcatcttc**agggcccgaggtgtgccagagaccttcaatgaagcc**aaggtcgcactgattgtcattcttctcttcgtgctgtcctgggctccctactccactgtggctctggtggcctttgctggatactcgcacatcctgacgccctacatgagctcggtgccagccgtcatcgccaaggcttctgccatccacaatcccattatctacgccatcact**caccccgagcagaacgtgcagaagcggaagcgcagcctcaagaagacctccacgatggcggccccgcccaagagcgagaactcagaggacgccaag**acggagactagccaggtggccccggccTAG

**>Gs**

ATGggctgcctcgggaacagtaagaccgaggaccagcgcaacgaggagaaggcgcagcgtgaggccaacaaaaagatcgagaagcagctgcagaaggacaagcaggtctaccgggccacgcaccgcctgctgctgctgggtgctggagaatctggtaaaagcaccattgtgaagcagatgaggatcctgcatgttaatgggtttaatggagagggcggcgaagaggacccgcaggctgcaaggagcaacagcgatggtgagaaggcaaccaaagtgcaggacatcaaaaacaacctgaaagaggcgattgaaaccattgtggccgccatgagcaacctggtgccccccgtggagctggccaaccccgagaaccagttcagagtggactacattctgagtgtgatgaacgtgcctgactttgacttccctcccgaattctatgagcatgccaaggctctgtgggaggatgaaggagtgcgtgcctgctacgaacgctccaacgagtaccagctgattgactgtgcccagtacttcctggacaagatcgacgtgatcaagcaggctgactatgtgccgagcgatcaggacctgcttcgctgccgtgtcctgacttctggaatctttgagaccaagttccaggtggacaaagtcaacttccacatgtttgacgtgggtggccagcgcgatgaacgccgcaagtggatccagtgcttcaacgatgtgactgccatcatcttcgtggtggccagcagcagctacaacatggtcatccgggaggacaaccagaccaaccgcctgcaggaggctctgaacctcttcaagagcatctggaacaacagatggctgcgcaccatctctgtgatcctgttcctcaacaagcaagatctgctcgctgagaaagtccttgctgggaaatcgaagattgaggactactttccagaatttgctcgctacactactcctgaggatgctactcccgagcccggagaggacccacgcgtgacccgggccaagtacttcattcgagatgagtttctgaggatcagcactgccagtggagatgggcgtcactactgctaccctcatttcacctgcgctgtggacactgagaacatccgccgtgtgttcaacgactgccgtgacatcattcagcgcatgcaccttcgtcagtacgagctgctcTGA

**>Gsi** (cysteine to serine mutation shown in red)

ATGggctgcctcgggaacagtaagaccgaggaccagcgcaacgaggagaaggcgcagcgtgaggccaacaaaaagatcgagaagcagctgcagaaggacaagcaggtctaccgggccacgcaccgcctgctgctgctgggtgctggagaatctggtaaaagcaccattgtgaagcagatgaggatcctgcatgttaatgggtttaatggagagggcggcgaagaggacccgcaggctgcaaggagcaacagcgatggtgagaaggcaaccaaagtgcaggacatcaaaaacaacctgaaagaggcgattgaaaccattgtggccgccatgagcaacctggtgccccccgtggagctggccaaccccgagaaccagttcagagtggactacattctgagtgtgatgaacgtgcctgactttgacttccctcccgaattctatgagcatgccaaggctctgtgggaggatgaaggagtgcgtgcctgctacgaacgctccaacgagtaccagctgattgactgtgcccagtacttcctggacaagatcgacgtgatcaagcaggctgactatgtgccgagcgatcaggacctgcttcgctgccgtgtcctgacttctggaatctttgagaccaagttccaggtggacaaagtcaacttccacatgtttgacgtgggtggccagcgcgatgaacgccgcaagtggatccagtgcttcaacgatgtgactgccatcatcttcgtggtggccagcagcagctacaacatggtcatccgggaggacaaccagaccaaccgcctgcaggaggctctgaacctcttcaagagcatctggaacaacagatggctgcgcaccatctctgtgatcctgttcctcaacaagcaagatctgctcgctgagaaagtccttgctgggaaatcgaagattgaggactactttccagaatttgctcgctacactactcctgaggatgctactcccgagcccggagaggacccacgcgtgacccgggccaagtacttcattcgagatgagtttctgaggatcagcactgccagtggagatgggcgtcactactgctaccctcatttcacctgcgctgtggacactgagaacatccgccgtgtgttcaacgactgccgtgat**gtcatcataaaaaataatctaaaagattctggtctctttTGA**

**>Gso** (cysteine to serine mutation shown in red)

ATGggctgcctcgggaacagtaagaccgaggaccagcgcaacgaggagaaggcgcagcgtgaggccaacaaaaagatcgagaagcagctgcagaaggacaagcaggtctaccgggccacgcaccgcctgctgctgctgggtgctggagaatctggtaaaagcaccattgtgaagcagatgaggatcctgcatgttaatgggtttaatggagagggcggcgaagaggacccgcaggctgcaaggagcaacagcgatggtgagaaggcaaccaaagtgcaggacatcaaaaacaacctgaaagaggcgattgaaaccattgtggccgccatgagcaacctggtgccccccgtggagctggccaaccccgagaaccagttcagagtggactacattctgagtgtgatgaacgtgcctgactttgacttccctcccgaattctatgagcatgccaaggctctgtgggaggatgaaggagtgcgtgcctgctacgaacgctccaacgagtaccagctgattgactgtgcccagtacttcctggacaagatcgacgtgatcaagcaggctgactatgtgccgagcgatcaggacctgcttcgctgccgtgtcctgacttctggaatctttgagaccaagttccaggtggacaaagtcaacttccacatgtttgacgtgggtggccagcgcgatgaacgccgcaagtggatccagtgcttcaacgatgtgactgccatcatcttcgtggtggccagcagcagctacaacatggtcatccgggaggacaaccagaccaaccgcctgcaggaggctctgaacctcttcaagagcatctggaacaacagatggctgcgcaccatctctgtgatcctgttcctcaacaagcaagatctgctcgctgagaaagtccttgctgggaaatcgaagattgaggactactttccagaatttgctcgctacactactcctgaggatgctactcccgagcccggagaggacccacgcgtgacccgggccaagtacttcattcgagatgagtttctgaggatcagcactgccagtggagatgggcgtcactactgctaccctcatttcacctgcgctgtggacactgagaacatccgccgtgtgttcaacgactgccgtgac**atcatcatcgccaaaaacctgcggggctctggactctactga**

**>Gst** (cysteine to serine mutation shown in red)

ATGggctgcctcgggaacagtaagaccgaggaccagcgcaacgaggagaaggcgcagcgtgaggccaacaaaaagatcgagaagcagctgcagaaggacaagcaggtctaccgggccacgcaccgcctgctgctgctgggtgctggagaatctggtaaaagcaccattgtgaagcagatgaggatcctgcatgttaatgggtttaatggagagggcggcgaagaggacccgcaggctgcaaggagcaacagcgatggtgagaaggcaaccaaagtgcaggacatcaaaaacaacctgaaagaggcgattgaaaccattgtggccgccatgagcaacctggtgccccccgtggagctggccaaccccgagaaccagttcagagtggactacattctgagtgtgatgaacgtgcctgactttgacttccctcccgaattctatgagcatgccaaggctctgtgggaggatgaaggagtgcgtgcctgctacgaacgctccaacgagtaccagctgattgactgtgcccagtacttcctggacaagatcgacgtgatcaagcaggctgactatgtgccgagcgatcaggacctgcttcgctgccgtgtcctgacttctggaatctttgagaccaagttccaggtggacaaagtcaacttccacatgtttgacgtgggtggccagcgcgatgaacgccgcaagtggatccagtgcttcaacgatgtgactgccatcatcttcgtggtggccagcagcagctacaacatggtcatccgggaggacaaccagaccaaccgcctgcaggaggctctgaacctcttcaagagcatctggaacaacagatggctgcgcaccatctctgtgatcctgttcctcaacaagcaagatctgctcgctgagaaagtccttgctgggaaatcgaagattgaggactactttccagaatttgctcgctacactactcctgaggatgctactcccgagcccggagaggacccacgcgtgacccgggccaagtacttcattcgagatgagtttctgaggatcagcactgccagtggagatgggcgtcactactgctaccctcatttcacctgcgctgtggacactgagaacatccgccgtgtgttcaacgactgccgtgac**atcatcatcaaggagaacctcaaagactctggcctcttctga**

**>Gsz**

ATGggctgcctcgggaacagtaagaccgaggaccagcgcaacgaggagaaggcgcagcgtgaggccaacaaaaagatcgagaagcagctgcagaaggacaagcaggtctaccgggccacgcaccgcctgctgctgctgggtgctggagaatctggtaaaagcaccattgtgaagcagatgaggatcctgcatgttaatgggtttaatggagagggcggcgaagaggacccgcaggctgcaaggagcaacagcgatggtgagaaggcaaccaaagtgcaggacatcaaaaacaacctgaaagaggcgattgaaaccattgtggccgccatgagcaacctggtgccccccgtggagctggccaaccccgagaaccagttcagagtggactacattctgagtgtgatgaacgtgcctgactttgacttccctcccgaattctatgagcatgccaaggctctgtgggaggatgaaggagtgcgtgcctgctacgaacgctccaacgagtaccagctgattgactgtgcccagtacttcctggacaagatcgacgtgatcaagcaggctgactatgtgccgagcgatcaggacctgcttcgctgccgtgtcctgacttctggaatctttgagaccaagttccaggtggacaaagtcaacttccacatgtttgacgtgggtggccagcgcgatgaacgccgcaagtggatccagtgcttcaacgatgtgactgccatcatcttcgtggtggccagcagcagctacaacatggtcatccgggaggacaaccagaccaaccgcctgcaggaggctctgaacctcttcaagagcatctggaacaacagatggctgcgcaccatctctgtgatcctgttcctcaacaagcaagatctgctcgctgagaaagtccttgctgggaaatcgaagattgaggactactttccagaatttgctcgctacactactcctgaggatgctactcccgagcccggagaggacccacgcgtgacccgggccaagtacttcattcgagatgagtttctgaggatcagcactgccagtggagatgggcgtcactactgctaccctcatttcacctgcgctgtggacactgagaacatccgccgtgtgttcaacgactgccgtgac**gtcatcatacagaacaatctcaagtacattggcctttgctga**

**>Gsq**

ATGggctgcctcgggaacagtaagaccgaggaccagcgcaacgaggagaaggcgcagcgtgaggccaacaaaaagatcgagaagcagctgcagaaggacaagcaggtctaccgggccacgcaccgcctgctgctgctgggtgctggagaatctggtaaaagcaccattgtgaagcagatgaggatcctgcatgttaatgggtttaatggagagggcggcgaagaggacccgcaggctgcaaggagcaacagcgatggtgagaaggcaaccaaagtgcaggacatcaaaaacaacctgaaagaggcgattgaaaccattgtggccgccatgagcaacctggtgccccccgtggagctggccaaccccgagaaccagttcagagtggactacattctgagtgtgatgaacgtgcctgactttgacttccctcccgaattctatgagcatgccaaggctctgtgggaggatgaaggagtgcgtgcctgctacgaacgctccaacgagtaccagctgattgactgtgcccagtacttcctggacaagatcgacgtgatcaagcaggctgactatgtgccgagcgatcaggacctgcttcgctgccgtgtcctgacttctggaatctttgagaccaagttccaggtggacaaagtcaacttccacatgtttgacgtgggtggccagcgcgatgaacgccgcaagtggatccagtgcttcaacgatgtgactgccatcatcttcgtggtggccagcagcagctacaacatggtcatccgggaggacaaccagaccaaccgcctgcaggaggctctgaacctcttcaagagcatctggaacaacagatggctgcgcaccatctctgtgatcctgttcctcaacaagcaagatctgctcgctgagaaagtccttgctgggaaatcgaagattgaggactactttccagaatttgctcgctacactactcctgaggatgctactcccgagcccggagaggacccacgcgtgacccgggccaagtacttcattcgagatgagtttctgaggatcagcactgccagtggagatgggcgtcactactgctaccctcatttcacctgcgctgtggacactgagaacatccgccgtgtgttcaacgactgccgtgac**accatcctccagttgaacctgaaggagtacaatctggtctga**

**>Gs12**

ATGggctgcctcgggaacagtaagaccgaggaccagcgcaacgaggagaaggcgcagcgtgaggccaacaaaaagatcgagaagcagctgcagaaggacaagcaggtctaccgggccacgcaccgcctgctgctgctgggtgctggagaatctggtaaaagcaccattgtgaagcagatgaggatcctgcatgttaatgggtttaatggagagggcggcgaagaggacccgcaggctgcaaggagcaacagcgatggtgagaaggcaaccaaagtgcaggacatcaaaaacaacctgaaagaggcgattgaaaccattgtggccgccatgagcaacctggtgccccccgtggagctggccaaccccgagaaccagttcagagtggactacattctgagtgtgatgaacgtgcctgactttgacttccctcccgaattctatgagcatgccaaggctctgtgggaggatgaaggagtgcgtgcctgctacgaacgctccaacgagtaccagctgattgactgtgcccagtacttcctggacaagatcgacgtgatcaagcaggctgactatgtgccgagcgatcaggacctgcttcgctgccgtgtcctgacttctggaatctttgagaccaagttccaggtggacaaagtcaacttccacatgtttgacgtgggtggccagcgcgatgaacgccgcaagtggatccagtgcttcaacgatgtgactgccatcatcttcgtggtggccagcagcagctacaacatggtcatccgggaggacaaccagaccaaccgcctgcaggaggctctgaacctcttcaagagcatctggaacaacagatggctgcgcaccatctctgtgatcctgttcctcaacaagcaagatctgctcgctgagaaagtccttgctgggaaatcgaagattgaggactactttccagaatttgctcgctacactactcctgaggatgctactcccgagcccggagaggacccacgcgtgacccgggccaagtacttcattcgagatgagtttctgaggatcagcactgccagtggagatgggcgtcactactgctaccctcatttcacctgcgctgtggacactgagaacatccgccgtgtgttcaacgactgccgtgac**accatcctgcaggagaacctgaaggacatcatgctgcagtga**

**>Gs13**

ATGggctgcctcgggaacagtaagaccgaggaccagcgcaacgaggagaaggcgcagcgtgaggccaacaaaaagatcgagaagcagctgcagaaggacaagcaggtctaccgggccacgcaccgcctgctgctgctgggtgctggagaatctggtaaaagcaccattgtgaagcagatgaggatcctgcatgttaatgggtttaatggagagggcggcgaagaggacccgcaggctgcaaggagcaacagcgatggtgagaaggcaaccaaagtgcaggacatcaaaaacaacctgaaagaggcgattgaaaccattgtggccgccatgagcaacctggtgccccccgtggagctggccaaccccgagaaccagttcagagtggactacattctgagtgtgatgaacgtgcctgactttgacttccctcccgaattctatgagcatgccaaggctctgtgggaggatgaaggagtgcgtgcctgctacgaacgctccaacgagtaccagctgattgactgtgcccagtacttcctggacaagatcgacgtgatcaagcaggctgactatgtgccgagcgatcaggacctgcttcgctgccgtgtcctgacttctggaatctttgagaccaagttccaggtggacaaagtcaacttccacatgtttgacgtgggtggccagcgcgatgaacgccgcaagtggatccagtgcttcaacgatgtgactgccatcatcttcgtggtggccagcagcagctacaacatggtcatccgggaggacaaccagaccaaccgcctgcaggaggctctgaacctcttcaagagcatctggaacaacagatggctgcgcaccatctctgtgatcctgttcctcaacaagcaagatctgctcgctgagaaagtccttgctgggaaatcgaagattgaggactactttccagaatttgctcgctacactactcctgaggatgctactcccgagcccggagaggacccacgcgtgacccgggccaagtacttcattcgagatgagtttctgaggatcagcactgccagtggagatgggcgtcactactgctaccctcatttcacctgcgctgtggacactgagaacatccgccgtgtgttcaacgactgccgtgac**actattctgcatgacaacctcaagcagcttatgctacagtga**

**>Gs15**

ATGggctgcctcgggaacagtaagaccgaggaccagcgcaacgaggagaaggcgcagcgtgaggccaacaaaaagatcgagaagcagctgcagaaggacaagcaggtctaccgggccacgcaccgcctgctgctgctgggtgctggagaatctggtaaaagcaccattgtgaagcagatgaggatcctgcatgttaatgggtttaatggagagggcggcgaagaggacccgcaggctgcaaggagcaacagcgatggtgagaaggcaaccaaagtgcaggacatcaaaaacaacctgaaagaggcgattgaaaccattgtggccgccatgagcaacctggtgccccccgtggagctggccaaccccgagaaccagttcagagtggactacattctgagtgtgatgaacgtgcctgactttgacttccctcccgaattctatgagcatgccaaggctctgtgggaggatgaaggagtgcgtgcctgctacgaacgctccaacgagtaccagctgattgactgtgcccagtacttcctggacaagatcgacgtgatcaagcaggctgactatgtgccgagcgatcaggacctgcttcgctgccgtgtcctgacttctggaatctttgagaccaagttccaggtggacaaagtcaacttccacatgtttgacgtgggtggccagcgcgatgaacgccgcaagtggatccagtgcttcaacgatgtgactgccatcatcttcgtggtggccagcagcagctacaacatggtcatccgggaggacaaccagaccaaccgcctgcaggaggctctgaacctcttcaagagcatctggaacaacagatggctgcgcaccatctctgtgatcctgttcctcaacaagcaagatctgctcgctgagaaagtccttgctgggaaatcgaagattgaggactactttccagaatttgctcgctacactactcctgaggatgctactcccgagcccggagaggacccacgcgtgacccgggccaagtacttcattcgagatgagtttctgaggatcagcactgccagtggagatgggcgtcactactgctaccctcatttcacctgcgctgtggacactgagaacatccgccgtgtgttcaacgactgccgtgac**tcggtgctcgcccgctacctggacgagatcaacctgctgtga**
